# Involvement of the Mitochondrial Protein Tyrosine Phosphatase PTPM1 in the Promotion of Conidiation, Development, and Pathogenicity in *Colletotrichum graminicola*

**DOI:** 10.3389/fmicb.2020.605738

**Published:** 2021-01-14

**Authors:** Shaowei Wang, Guihua Li, Yi Wei, Gang Wang, Yuejia Dang, Penghui Zhang, Shi-Hong Zhang

**Affiliations:** ^1^College of Plant Sciences, Jilin University, Changchun, China; ^2^Key Laboratory of Zoonosis Research, Ministry of Education, Jilin University, Changchun, China; ^3^College of Plant Protection, Shenyang Agricultural University, Shenyang, China; ^4^School of Life Sciences, Henan University, Kaifeng, China

**Keywords:** mitochondrial protein tyrosine phosphatase (CgPTPM1), *Colletotrichum graminicola*, pathogenicity, conidiation, reactive oxygen species

## Abstract

The phosphorylation status of proteins, which is determined by protein tyrosine kinases (PTKs) and protein tyrosine phosphatases (PTPs), governs many cellular actions. In fungal pathogens, phosphorylation-mediated signal transduction has been considered to be one of the most important mechanisms in pathogenicity. *Colletotrichum graminicola* is an economically important corn pathogen. However, whether phosphorylation is involved in its pathogenicity is unknown. A mitochondrial protein tyrosine phosphatase gene, designated *CgPTPM1*, was deduced in *C. graminicola* through the use of bioinformatics and confirmed by enzyme activity assays and observation of its subcellular localization. We then created a *CgPTPM1* deletion mutant (Δ*CgPTPM1*) to analyze its biological function. The results indicated that the loss of *CgPTPM1* dramatically affected the formation of conidia and the development and differentiation into appressoria. However, the colony growth and conidial morphology of the Δ*CgPTPM1* strains were unaffected. Importantly, the Δ*CgPTPM1* mutant strains exhibited an obvious reduction of virulence, and the delayed infected hyphae failed to expand in the host cells. In comparison with the wild-type, Δ*CgPTPM1* accumulated a larger amount of H_2_O_2_ and was sensitive to exogenous H_2_O_2_. Interestingly, the host cells infected by the mutant also exhibited an increased accumulation of H_2_O_2_ around the infection sites. Since the expression of the *CgHYR1*, *CgGST1*, *CgGLR1*, *CgGSH1* and *CgPAP1* genes was upregulated with the H_2_O_2_ treatment, our results suggest that the mitochondrial protein tyrosine phosphatase PTPM1 plays an essential role in promoting the pathogenicity of *C. graminicola* by regulating the excessive *in vivo* and *in vitro* production of H_2_O_2_.

## Introduction

As a whole, phosphorylation and dephosphorylation regulate physiological processes, such as gene expression, signal transduction and the cell cycle, in eukaryotic cells (Zolnierowicz and Bollen, 2000; Cohen, 2001; Tonks, 2013). The reversible phosphorylation process, an important regulatory mechanism, is regulated by more than 500 protein kinases and 100 protein phosphatases (PPs) (Manning et al., 2002; Alonso et al., 2004). Protein kinases can phosphorylate serine (Ser), threonine (Thr) or tyrosine (Tyr) residues to form active phosphorylated proteins, and alternatively, PPs can remove the phosphate group from the phosphorylated protein (Guan et al., 1990; Hunter, 1995). PPs can be divided into serine/threonine phosphatases and tyrosine phosphatases (PTPs) based on their sequence homology, specificity of catalytic substrates and different conformations of autocatalytic regions (Keyse, 1995; Pao et al., 2007; Shi, 2009). Typical PTPs and DUSPs (dual-specificity phosphatases) have similar catalytic mechanisms in the hydrolysis of phosphorylated substrates (Denu and Dixon, 1995, 1998). They share a highly conserved catalytic domain, which has a signature motif HCXXGXXR that forms the catalytic active pocket known as the P-loop (Tu et al., 2020). Cysteine (Cys) plays the main catalytic role in this motif, while arginine (Arg) plays a coordinated role during the catalytic process (Patterson et al., 2009; Tonks, 2013). Studies have shown that when Cys is replaced with Ser, the enzyme will lose its hydrolytic activity *in vitro* (Ishibashi et al., 1992; Sun et al., 1993), and its function *in vivo* will be inhibited (Sun et al., 1993; Doi et al., 1994; Aroca et al., 1995).

The current research on PTPs is concentrated in humans and mammals and has shown that PTPs are closely related to morphological development, signal transmission and disease generation (Barford et al., 1994; Alonso et al., 2004). Moreover, studies have shown that PTPs have a regulatory effect on numerous aspects of fungi, such as development, meiosis and pathogenicity. For example, in *Saccharomyces cerevisiae*, the PTPs PTP2 and PTP3 can affect meiosis and spore formation (Zhan et al., 2000). DUSP Rok1 regulates the mating and toxicity functions of *Ustilago maydis* by its control of the levels of phosphorylation of Kpp2 and Kpp6 with the MAPKs (mitogen-activated protein kinases) signal pathway (Di Stasio et al., 2009). Most of the 14 PTPs in *Neurospora crassa* are related to the growth of mycelia and spore formation (Ghosh et al., 2014), while in *Magnaporthe oryzae*, MoPTP2, the homolog of PTP2 in yeast, plays a role in the resistance to fungicide fludioxonil. It participates as a key factor in the regulation of high-osmolarity glycerol pathway (Bohnert et al., 2019).

Mitochondria, dynamic double-membrane organelles in eukaryotic cells, can provide most of the energy required for the survival of organisms through oxidative phosphorylation and electron transport chains. In addition to their role in providing energy, more evidence shows that the mitochondria participate in physiological processes, such as metabolism, cell-cycle control, development and cell death, through intracellular signal transduction processes. In these processes, many types of proteins, such as GTPases, kinases and phosphatases, are all involved (McBride et al., 2006). However, to our knowledge, the current research on mitochondrial phosphatase is primarily concentrated in mammals and yeast (Osman et al., 2010; Zhang et al., 2011), while research in filamentous fungi has rarely been reported. In addition, some studies have indicated that mitochondria are the main source of reactive oxygen species (ROS) in organisms (Friguet, 2006; Zhang et al., 2011). It is well known that appropriate levels of ROS in animals, plants and fungi can be used as important signal molecules to participate in many physiological regulation processes. For example, in *M. oryzae*, two NADPH oxidases, NOX1 and NOX2, play a role in the maturation of appressoria, as well as in pathogenicity (Thannickal and Fanburg, 2000; Egan et al., 2007). However, excessive ROS will cause the aging and death of cells, which causes many diseases (Yang et al., 2015). During the long process of evolution, plants have evolved the function of using ROS to resist the invasion of pathogens, and the production of ROS can also be regarded as the first line of defense for plants to resist these incursions (Apostol et al., 1989; Doke et al., 1996; Mellersh et al., 2002). Whether the pathogen can effectively remove the exogenous ROS is also the key to its success in infecting the host (Mayer et al., 2001; Apel and Hirt, 2004; Egan et al., 2007). Studies have shown that many virulence factors of pathogenic fungi also have antioxidant functions (Ding et al., 2010; Cui et al., 2015; Li et al., 2015). In addition, the cell wall integrity (CWI) regulated by the CWI MAPK signaling pathway also plays an important role in growth and pathogenesis in filamentous ascomycetes (Li et al., 2012). For example, the presumed DUSP MoYvh1 can affect the CWI, sensitivity to ROS and pathogenicity in *M. oryzae* (Liu et al., 2016), while the serine/threonine phosphatase MoPpe1 reduces the CWI by interacting with its functionally similar MoSap1, resulting in a decrease in the pathogenicity of *M. oryzae* (Qian et al., 2018).

Corn anthracnose is a disease caused by the hemibiotrophic filamentous fungus *Colletotrichum graminicola* (Ces.) G.W. Wils., and the common forms of this disease are anthracnose leaf blight and anthracnose stalk rot (Bergstrom and Nicholson, 1999; Munch et al., 2008; Dean et al., 2012), which dramatically affects the production of global corn each year (Frey et al., 2011; Miranda et al., 2017). The falcate conidia are the main mechanism for its infection (Bergstrom and Nicholson, 1999; Wang et al., 2016). When the conidia germinate on the surface of the host, they will form specialized infection cells at the tip of the germ tube, called appressoria (Mercure et al., 1994; Bergstrom and Nicholson, 1999). A large volume of hypertonic substances such as glycerin will accumulate in the appressorium, and its turgor pressure can reach 5.4 MPa. Under the action of turgor pressure, the front of the appressorium will form a penetration peg, which can penetrate the epidermal cell of the host (Deising et al., 2000; Ludwig et al., 2014). After successful infection, the fungus enters a short biotrophic stage, during which the infection vesicle and primary hyphae formed do not cause macroscopic visible damage to the host cells. Next, on the basis of the primary hyphae, secondary hyphae are formed to penetrate the host plasma membrane and secrete degradative enzymes, which lead to cell death, and the pathogen enters the necrotrophic stage in which it will form obvious lesions on the host surface (Bergstrom and Nicholson, 1999; Munch et al., 2008). Although the physiological characteristics and pathogenicity of *C. graminicola* have been studied, the molecular mechanism behind it has yet to be explored in detail.

In this study, we deduced the existence of protein tyrosine phosphatase CgPTPM1 in *C. graminicola* and obtained the deletion and complementation strains of *CgPTPM1*. Biological phenotype tests showed that the mutant produced fewer conidia, reduced its rate of conidial germination, formed fewer appressoria and decreased its cell wall integrity. The pathogenicity of Δ*CgPTPM1* was significantly reduced, and the expansion of the infected hyphae in the host cells was inhibited. In addition, *CgPTPM1* is associated with the regulation of excessive amounts of H_2_O_2_. The expression of *CgPTPM1* can also upregulate the levels of expression of a series of genes related to H_2_O_2_ scavenging. To our knowledge, there are few studies on PTPs in *C. graminicola*. Therefore, our research has important scientific significance for exploring the role of PTPs in the physiology and pathogenicity of this virulent fungus.

## Materials and Methods

### Strains and Culture Conditions

The *C. graminicola* M1.001 strain used as wild type (WT) in this study was a gift from Dr. Holger B. Deising (Institute of Agricultural and Nutritional Sciences, Martin Luther University Halle-Wittenberg, Halle, Germany). The wild type, mutant and complementation strains were cultured at 25°C. Potato dextrose agar (PDA: 200 g/L peeled potatoes, 20 g/L glucose and 15 g/L agar) was used for the growth and conidial production of the strains. Phenotypic tests were performed on complete minimal media (CM: 1 g/L yeast extract, 0.5 g/L enzyme hydrolyzed casein, 0.5 g/L acid hydrolyzed casein, 10 g/L glucose, 1 g/L Ca(NO_3_)_2_⋅4H_2_O, 0.2 g/L KH_2_PO_4_, 0.25 g/L MgSO_4_⋅7H_2_O, 0.15 g/L NaCl and 15 g/L agar). All the fungal strains used were maintained on paper filters at −20°C. *Agrobacterium tumefaciens* AGL-1 and *Escherichia coli* strains DH5α and BL21 were cultured in lysogeny broth media (LB: 10 g/L peptone, 5 g/L yeast extract and 10 g/L NaCl, pH 7.0).

### Bioinformatics Analysis

The required nucleotide and protein sequences were downloaded and analyzed from the NCBI^1^ databases and Ensembl-Genomes-Database^2^. The subcellular localization of CgPTPM1 in *C. graminicola* was predicted using the online software program ProtComp 9.0^3^. The mitochondrial targeting sequence of CgPTPM1 was predicted using the online software MitoProtII^4^. The analysis of protein domain was performed using the online software SMART^5^. The multi-sequence alignment of amino acid sequences was conducted using the software DNAMAN (Lynnon Biosoft, San Ramon, CA, United States), and the alignment results were used to generate phylogenetic trees using the adjacent tree method of software MEGA 7.0^6^. The prediction of the three-dimensional structure of protein was conducted using the SWISS-MODEL server^7^, and the results from prediction were processed and drawn using the software PyMOL (DeLano Scientific, LLC, Palo Alto, CA, United States). The predicted molecular weight of the protein was calculated using the online software program ExPASy^8^.

### Nucleic Acid Isolation and Polymerase Chain Reaction

The mycelia used for nucleic acid extraction were prepared by collecting the relevant strains cultivated in 100 mL liquid CM media at 25°C and 150 rpm for 3 days. The collected mycelia were then washed using ddH_2_O and desiccated. The cetyltrimethylammonium bromide method was used to extract the genomic DNA. The primers were designed using Primer Premier 5.0 (Premier Biosoft International, San Francisco, CA, United States). The polymerase chain reaction (PCR) used high-fidelity polymerase (TaKaRa, Dalian, China) for amplification. All the PCR fragments were sequenced by Sangon Biotech Company (Shanghai, China). The total RNA was extracted using the reagent RNAiso Plus (TaKaRa), and the RNA concentration was measured using a microspectrophotometer (Implen NanoPhotometer-N50, München, Germany). The first strand cDNA was synthesized by the reverse transcription-polymerase chain reaction using the TaKaRa Reverse Transcription Kit. The reverse transcribed cDNA was used for quantitative real-time PCR (qRT-PCR) by its reaction in an Applied Biosystems 7500 Real-Time PCR system (Foster City, CA, United States) and SYBR^®^ Premix EX Taq^TM^II (TaKaRa) using primers Gq-YP-F and Gq-YP-R (Supplementary Table S1). In qRT-PCR, an actin gene (GLRG_03056) with stable expression was used as a reference for normalization using primers Gq-actin-F and Gq-actin-R (Supplementary Table S1). The level of expression of messenger RNA was calculated using the 2^−ΔΔ*Ct*^ method (Livak and Schmittgen, 2001).

### Expression, Purification, Determination of Activity, and Subcellular Localization of the CgPTPM1 Protein

Using primers GLRG-BD-F and GLRG-BD-R (Supplementary Table S1) to amplify a 1869 bp fragment with *Hind*III and *Xho*I restriction sites from the cDNA of wild type M1.001, and the fragment was ligated to vector pET28a using a His-tag to generate an expression recombined vector. The recombined vector was then introduced into competent *E. coli* BL21 cells using heat shock transformation, and 1 mM isopropyl-ß-D-thiogalactopyranoside was used to induce the expression of the fusion protein at 25°C. After 8 h, the bacteria were pelleted by centrifugation at 5000 rpm for 20 min at 4°C, and the cells were resuspended by adding lysis buffer (50 mM Tris, 300 mM NaCl, 5 mM imidazole and 5 mM mercaptoethanol, pH 8.0) for ultrasonication. After centrifugation of the product at 5000 rpm at 4°C, the supernatant was passed through a Ni-NTA purification resin prepacked column (Sangon Biotech, Shanghai, China) to bind the fusion protein to the resin. The fusion protein was eluted and purified using 250 mM imidazole, and then it was dialyzed through dialyzate buffer (50 mM Tris, 300 mM NaCl, 5 mM mercaptoethanol, pH 8.0) and detected on a 12% SDS-PAGE gel. The concentration of the purified protein was measured using a bicinchoninic acid kit (Beyotime, Shanghai, China) (Liu et al., 2004; Wu et al., 2004).

pNPP (*p*-nitrophenyl phosphate) is a substrate that is commonly used to measure the activity of phosphatase (Zhang and VanEtten, 1991; Tonks, 2013). The phosphatase activity of purified CgPTPM1 was assayed with slight modifications as described by Dunphy and Kumagai (1991). Simultaneously, standard chicken intestinal alkaline phosphatase (Sangon Biotech, Shanghai, China) was used as a positive control, and heat-treated inactivated CgPTPM1 was used as a negative control. The specific steps were as follows: 10 μg of purified protein was added to a total volume of 100 μL of reaction buffer (0.1 M Tris, 0.25 M NaCl, 5 mM EDTA and 10 mM DTT, pH 8.2) with a defined concentration of pNPP. The reaction was incubated at 37°C for 30 min and terminated by the addition of 2 μL of 10 M NaOH. The absorbance of different color products was measured using a spectrophotometer (Implen NanoPhotometer-N50) at 405 nm using the reaction buffer lacking pNPP as a blank control. Subsequently, the measurement curve to determine the activity of the enzyme was drawn based on the substrate concentration and absorbance. This experiment was performed in triplicate.

The conidia and hyphae of the subcellular localization strains were observed using a fluorescence inverted microscope (Nikon ECLIPSE Ts2R, Tokyo, Japan) to observe the localization of CgPTPM1 in *C. graminicola*.

### Generation of *CgPTPM1* Deletion, Subcellular Localization, and Complementation Strains

The deletion mutants were obtained by replacing *CgPTPM1* with the 1.4-kb hygromycin B phosphotransferase gene (*HPH*) cassette from the vector pXEH 2.0 (Supplementary Figure 2A). First, the primer pairs GLRG-qc-LF/GLRG-qc-LR and GLRG-qc-RF/GLRG-qc-RR (Supplementary Table S1) were used to amplify the 955 bp upstream and 1011 bp downstream flanking fragments from wild type genomic DNA, respectively. The flanking fragments were treated with restriction enzymes and ligated to the *Eco*RI- *Kpn*I and *Xba*I*- Sal*I sites of the pXEH 2.0 vector to construct a recombinant vector, which was transformed into *A. tumefaciens* (AGL-1) competent cells. The AGL-1 strain was transformed with wild-type M1.001 using an *A. tumefaciens*-mediated transformation (ATMT) protocol (Munch et al., 2011). Transformants were selected on PDA plates with 100 μg/mL HygB, and three pairs of specific primers CgPTPM1-F/CgPTPM1-R, HYG-YZ-F/HYG-YZ-R and G-L-F/G-L-R (Supplementary Table S1) were used to detect the genomes of transformants (Supplementary Figure 2C). In addition, the levels of expression of the gene deletion strains were determined by qRT-PCR to further verify the results (Supplementary Figure 2D).

To obtain the subcellular localization strain of *CgPTPM1*, the cDNA of M1.001 was amplified using primers CgPTPM1HB-F and CgPTPM1HB-R (Supplementary Table S1) to obtain the target fragment without an intron and the stop codon. This fragment was ligated to the *Sma*I site of pKD7-Red vector (a gift from Dr. Hongkai Wang and Dr. Jianping Lu, Zhejiang University, Hangzhou, China) to construct the recombinant vector pKD7-Red*-CgPTPM1* (Supplementary Figure 3B), which was introduced into AGL-1 competent cells. The target fragment was inserted into the genome of deletion mutant using an ATMT protocol. Transformants were screened on PDA plates that contained 300 μg/mL G418, and the fusion fragments in the genome of positive transformants were verified with primers GLRG-HB-Red-F and GLRG-HB-Red-R (Supplementary Table S1, Supplementary Figure 3Ca). Moreover, the level of expression of the subcellular localization strain was detected using qRT-PCR (Supplementary Figure 3Cb).

For complementation, a 3642 bp fragment containing a 1119 bp upstream sequence, a full-length *CgPTPM1* gene coding region, and a 597 bp downstream sequence, was amplified from the wild-type genomic DNA using the primer pair G-hb-F/G-hb-R. The fragment was ligated to the *Hind*III and *Kpn*I sites of the pCB1532 vector (Supplementary Figure 4B). The recombinant vector were introduced into the *CgPTPM1* gene deletion strain using ATMT method as above. Complementation transformants were screened on DCM (Yeast nitrogen without amino acid 1.7 g/L, L-asparagine 2 g/L, glucose 10 g/L, NH_4_NO_3_ 1 g/L, Na_2_HPO_4_ used to adjust pH to 6.0) supplemented with 100 μg/mL Chlorimuron-ethyl and were confirmed using PCR and qRT-PCR (Supplementary Figure 4C).

### Analysis of *CgPTPM1* on the Growth, Conidiation, Conidial Germination, and Formation of Appressoria

The vegetative growth rate was determined by measuring the colony diameter on CM plates 7 days after the inoculation of 5 mm mycelial plugs of the wild type, mutant and complementation strains at 25°C. Conidiation was assayed by culturing three types of strains on PDA plates for 14 days at 25°C with continuous incandescent lighting to induce the formation of falcate conidia. The conidia were harvested using 10 mL of distilled water and filtered through three layers of lens paper. The conidia were centrifuged at 5000 rpm for 5 min and resuspended in 5 mL of distilled water. The numbers of conidia in 10 μL of the conidial suspension were counted using an optical microscope (Olympus, Tokyo, Japan). In addition, when the strains were induced to sporulate on the PDA plates, 20 mm × 5 mm colonies were selected to observe the conidiation directly under an optical microscope. To detect the difference in the conidial germination and appressorial formation among different types of strains, 30 μL of 1 × 10^5^ conidia/mL suspensions each from the wild type, mutant and complementation strains were placed separately on the surfaces of 4-week-old corn leaves, artificial hydrophobic films or onion epidermal cells. The samples were incubated at 90% humidity in a 25°C incubator in the dark. The germination of more than 200 conidia was observed under an optical microscope every 8 h, and the appressorial formation of more than 200 conidia was observed after 24 h. These experiments were performed in triplicate and repeated three times for each strain.

### Pathogenicity Assays

The corn cultivar used in the pathogenicity experiment was “Xianyu 335,” which is a variety that is planted widely in north China. The leaf spot inoculation test utilized 10 μL 5 × 10^5^ conidia/mL (2% gelatin, w/v) droplets that were placed separately on the surfaces of third corn leaves. After inoculation, the leaves were placed in an artificial climate box with a relative humidity of 90% at 25°C and incubated in the dark for the first 24 h and then cultivated continuously for 6 days in the dark followed by light for 12 h each before examination. In the pathogenicity test of the fungus for whole plant, 5 mL conidial suspensions at a concentration of 1 × 10^5^ conidia/mL were sprayed evenly on 4-week-old corn plants. The subsequent processing was the same as the leaf spot inoculation test. The disease of the whole plant was observed and photographed after 7 days.

The leaves after spot inoculation were sampled at 24 h, 48 h and 72 h, respectively, to microscopically observe the infection process. The samples were then sliced and observed for pathogenicity with an optical microscope. A statistical analysis was conducted with four different pathogenic types at 72 h post inoculation (hpi), and a total of more than 200 infectious structures were counted. These experiments were performed in triplicate and repeated three times for each strain.

### Stress Treatments and Cell Wall Integrity Test

To detect the effect of *CgPTPM1* under exogenous H_2_O_2_, different types of strains were continuously cultured on CM plates with concentrations of 0 mM, 1 mM, 2 mM and 3 mM H_2_O_2_ in the dark for 7 days at 25°C, and the fungal colonies were observed and measured. NaCl and sorbitol at concentrations of 2.5% and 5% (w/v), respectively, were added to the CM plates to test the degree of osmotic stress. After inoculation, the growth of fungal colonies was observed, and the diameters of colonies were measured after 7 days. Five millimeter mycelial plugs were inoculated on CM plates with Congo red (CR) of 100 or 200 μg/mL, and SDS of 0.005% or 0.01% (w/v), respectively, and cultured in the dark at 25°C for 7 days to test the integrity of the cell wall. The growth of the colonies was observed, and the diameters were measured. Both CR and SDS can interfere with fungal cell wall assembly (Woldringh and van Iterson, 1972; Roncero and Duran, 1985; Bruno et al., 2006). These experiments were performed in triplicate and repeated three times for each strain.

### DAB Staining Assay and Endogenous H_2_O_2_ Measurements

The 3,3′-diaminobenzidine (DAB) reagent can be used to detect the accumulation of H_2_O_2_. DAB is converted to an insoluble brown end-product in the presence of H_2_O_2_. To test the ability of *CgPTPM1* to clear the ROS produced by the host during the pathogenic process, leaves at 48 h that had been inoculated with a concentration of 1 × 10^5^ conidia/mL were sliced and stained in DAB dye solution (pH 3.8) of 1 mg/mL for 8 h and destained with ethanol/acetic acid (94:4, v/v) for 1 h (Marroquin-Guzman et al., 2017). The decolored samples were observed with an optical microscope. To detect the accumulation of endogenous H_2_O_2_ in the fungus, the mycelia of wild type, mutant and complementation strains cultured on CM plates for 4 days were collected and incubated in a 1 mg/ml solution of DAB at 25°C in the dark for 8 h. The samples were observed under an optical microscope after washes with distilled water.

The endogenous H_2_O_2_ content in *C. graminicola* was measured as previously described for plants (Brennan and Frenkel, 1977). The H_2_O_2_ was extracted from the wild type, mutant and complementation strains by homogenizing 3 g of mycelia in 6 mL of cold acetone. The homogenate was centrifuged at 3500 *g* for 5 min at room temperature, and the resulting supernatant was designated as the sample extract. Subsequently, 0.1 mL titanium reagent (5% [w/v] titanic sulfate in concentrated H_2_SO_4_) was added to 1 mL of the sample extract followed by the addition of 0.2 mL strong aqueous ammonia to precipitate the peroxide-titanium complex. The precipitated sample was centrifuged at 3000 *g* for 10 min at room temperature, and the supernatant was discarded. A volume of 5 mL of 2 M H_2_SO_4_ was added to completely solubilize the precipitate. The absorbance of the samples was measured at 415 nm with 2 M H_2_SO_4_ as a blank control.

The content of H_2_O_2_ in the sample extract was calculated using a standard curve. Reagents were added to seven 15 mL centrifuge tubes in sequence as described in Supplementary Table S2 and centrifuged at 3000 *g* for 10 min. The supernatant was discarded. A volume of 2 M H_2_SO_4_ was added to completely dissolve the precipitate and diluted to 6 mL with H_2_SO_4_. The absorbance at 415 nm was measured and used to draw a standard curve (Supplementary Figure 8B). The content of H_2_O_2_ in mycelia (*Y*) was calculated using the following equation:

$$Y=\frac{C\times V\,t}{F\,W\times V\,1}$$

where *C* is the H_2_O_2_ content calculated by the standard curve; *Vt* is the total volume of the sample extract; *FW* represents the fresh weight of mycelia, and *V1* is the volume of the sample extract used in the determination. These experiments were performed in triplicate and repeated three times for each strain.

### The Pattern of Expression of CgPTPM1 in Different Stages and Analysis of the Levels of Expression of ROS-Related Genes

We collected samples of the wild type strain in different stages, including vegetative growth, conidial production, formation of germ tube, appressorial formation and the pathogenic stage, for total RNA extraction to detect the pattern of expression of *CgPTPM1* using qRT-PCR. Moreover, to test the effect of *CgPTPM1* on other genes related to ROS scavenging, we cultured the wild type, mutant and complementation strains in liquid CM media without H_2_O_2_ or with a concentration of 4 mM H_2_O_2_. After 5 days of continuous cultivation at 25°C in the dark, the mycelia were collected for RNA extraction and reverse transcribed into cDNA. The relative expression levels of the following genes *CgPTPM1* (GLRG_07262), *CgHYR1* (GLRG_03111), *CgGST1* (GLRG_06829), *CgGLR1* (GLRG_06921), *CgGSH1* (GLRG_03334), and *CgPAP1* (GLRG_05388) were measured with primers (Supplementary Table S1) using qRT-PCR. The experiment was conducted three times with three independent biological replicates for each strain.

### Statistical Analysis

All quantitative data provided in this study represent the results of triplicate experiments independently performed at least three times. Origin 7.0 software (OriginLab Corp., Northampton, MA, United States) was used to analyze the data and determine the mean ± SD of enzyme activity, conidiation, rate of conidial germination, rate of appressorial formation, colony diameters, relative expression and different types of disease. The significance of the data was assessed using the Student’s *t*-test. *P* < 0.05 was considered statistically significant. Error bars represent the standard deviation.

## Results

### CgPTPM1 Is a Mitochondrial Protein Tyrosine Phosphatase in *C. graminicola*

**FIGURE 1:**
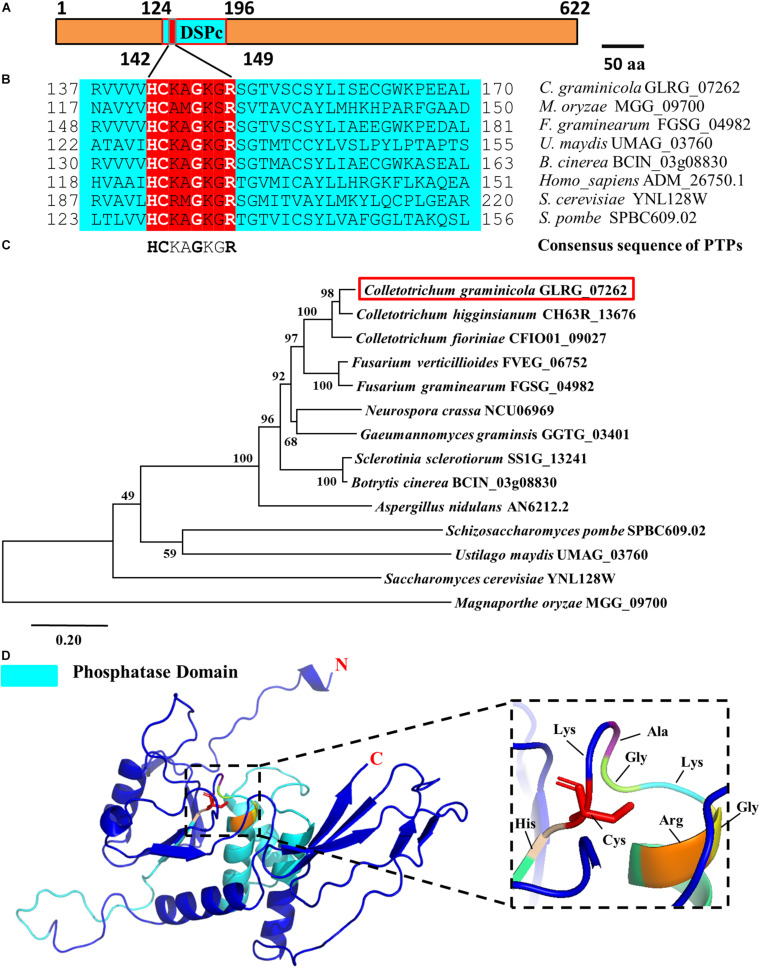
Bioinformatics analysis of the *CgPTPM1* gene in *C. graminicola*. **(A)** A schematic representation of the domain architecture of CgPTPM1. The phosphatase domain of CgPTPM1 is from position 124 to 196, and the amino acid sequences from positions 142 to 149 are the catalytic core motif. The essential Cys residue is located at position 143. **(B)** Comparative analysis of conserved regions in the protein. The catalytic domain of CgPTPM1 is compared with the phosphatase catalytic domain sequences that have been reported in other species. These species include *Saccharomyces cerevisiae* S288C, *Schizosaccharomyces pombe*, *Homo sapiens*, *Magnaporthe oryzae* 70-15, *Fusarium graminearum* PH-1, *Ustilago maydis* 521 and *Botrytis cinerea* B05.10. The red box marks the phosphatase catalytic core motifs in different species. **(C)** Phylogenetic tree of protein CgPTPM1 (GLRG_07262, marked in red). The phylogenetic tree represents the genetic relationship between CgPTPM1 and its homologous proteins in different species. An analysis of homologous amino acid sequence alignments was performed using the MUSCLE program, and a phylogenetic tree was constructed using the Neighbor-Joining algorithm. **(D)** Prediction of the spatial structure of protein CgPTPM1. The online software SWISS-MODEL was used to predict the three-dimensional structure of the protein, and PyMOL was used to organize and map the content. The cyan area in the structure represents the phosphatase domain. The enlarged part is the catalytic core motif, including 8 amino acid residues that are marked.

In different species, PTPs includes two domains, namely the N-terminal domain and the C-terminal domain. The N-terminal catalytic domain is more conserved, while the C-terminal domain varies in different types of PTPs. Structural biological studies have shown that PPs can be divided into three branches based on the different HC(X)_5_R sequences during the evolution process, and the active motif of PTPs is HCXXGXXR (Denu and Dixon, 1995, 1998). Based on the conserved characteristics of the catalytic domain of PTPs and combined with the existing research results in filamentous fungi, we compared the databases of *C. graminicola* in the NCBI and Ensembl-Genomes-Database and found a putative PTP in which the coding gene is GLRG_07262. Subcellular localization prediction showed that the protein is primarily distributed on the mitochondria (Table 1), and actually, a predicted mitochondrial targeting sequence “MASLLRQIVAGPRAR” had been found in the protein. Therefore, we designated the protein CgPTPM1. A bioinformatics analysis showed that the protein contained 622 amino acid residues, and the predicted molecular weight was approximately 70 kDa. The full length of the open reading frame was 1926 bp, and it contained two exons of 98 bp and 1771 bp and an intron of 57 bp. The analysis indicated that the N-terminal 124-196 sequences of CgPTPM1 were an active region of PTPs, which contained the conserved catalytic motif HCKAGKGR at positions 142-149, and the essential Cys residue was located at position 143 (Figure 1A). By comparing the amino acid sequence of CgPTPM1 with the PTPs sequences of the reported species, it was found that the core catalytic motifs, i.e., HCXXGXXR, were highly conserved among various species (Figure 1B). A phylogenetic tree analysis showed that CgPTPM1 had a close relationship with other homologs in fungi (Figure 1C). The 3D modeling result showed that CgPTPM1 had no ligands; the catalytic domain and active sites were located inside the protein structure and surrounded by multiple α-helices (Figure 1D).

**TABLE 1:** Prediction of subcellular localization of CgPTPM1 in *C. graminicola.*

Predicted by Neural nets: Mitochondrial with score: 1.08. Predicted by Pentamers: Mitochondrial with score: 1.2. Integral Prediction of protein location: Mitochondrial with score: 5.6.					

Location weights:	LocDB	PotLocDB	Neural nets	Pentamers	Integral
Nuclear	0.0	0.0	0.40	0.0	0.31
Plasma membrane	0.0	0.0	0.34	0.21	1.97
Extracellular	0.0	0.0	0.26	0.35	1.16
Cytoplasmic	0.0	0.0	0.24	0.06	0.29
Mitochondrial	0.0	0.0	1.08	1.2	5.60
Endoplasm. retic.	0.0	0.0	0.28	0.02	0.17
Peroxisomal	0.0	0.0	0.02	0.09	0.00
Lysosomal	0.0	0.0	0.09	0.02	0.00
Golgi	0.0	0.0	0.00	1.08	0.36
Vacuolar	0.0	0.0	0.29	0.05	0.13

*LocDB: scores based on query protein’s homologies with proteins of known localization.*

*PotLocDB: scores based on homologies with proteins which locations are not experimentally known but are assumed from strong theoretical evidence.*

*NNets: scores assigned by neural networks.*

*Pentamers: scores based on comparisons of pentamer distributions calculated for QUERY and DB sequences.*

*Integral are final scores that combine all above previous scores using a final neural net.*

*http://www.softberry.com/berry.phtml?topic=protcompan&group=help&subgroup=proloc*

**FIGURE 2:**
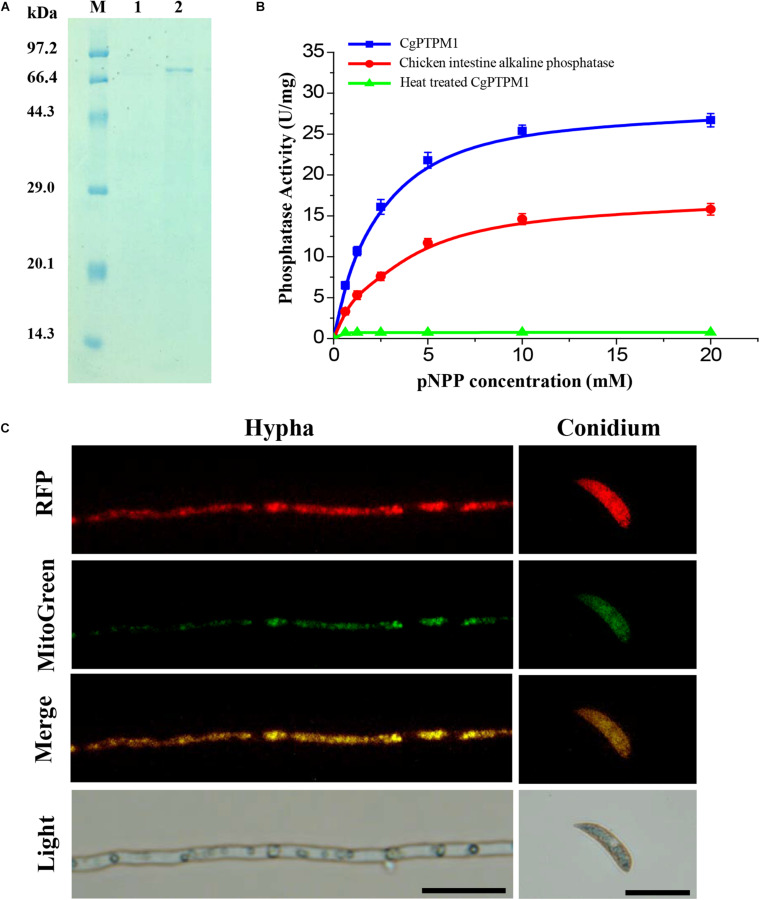
Expression and purification, phosphatase activity test, and subcellular localization of the CgPTPM1 protein. **(A)** SDS-PAGE analysis of CgPTPM1. Lane M: protein marker (TaKaRa, Dalian, China). Lane 1: the negative control without induction of isopropyl-ß-D-thiogalactopyranoside (IPTG). Lane 2: expression and purification induced by 1 mM IPTG. **(B)** Phosphatase activity test. The enzyme activity was measured using pNPP as a substrate. The standard chicken intestine alkaline phosphatase was used as the positive control, and the inactivated CgPTPM1 served as the negative control. As the substrate concentration increased, the enzymatic reaction rate increased, and the data conformed to the Michaelis-Menten equation. Error bars represent ± SD of three independent repeated samples. **(C)** Subcellular localization of CgPTPM1 in *C. graminicola*. The CgPTPM1-RFP fusion protein was used to observe the distribution of CgPTPM1, and the green fluorescent dye MitoTracker Green^®^ FM was used to label the mitochondria in the cells. In the merged image, the original red fluorescence from CgPTPM1-RFP colocalized with the green fluorescence Mito-Green appearing as yellow areas. Scale bar = 20 μm.

We constructed a prokaryotic expression bacterium of *CgPTPM1* and obtained a purified protein with a size of approximately 74 kDa (Figure 2A), and the concentration of the protein was 0.2 mg/mL. Using pNPP as a substrate, the protein phosphatase activity was detected using the colorimetric method at 405 nm. The results showed that the enzymatic reaction rate gradually increased in parallel with that of the substrate concentration, and the specific activity of CgPTPM1 was higher than that of the standard product (Figure 2B), thus, proving that CgPTPM1 had phosphatase activity.

To understand the function of CgPTPM1, it is essential to determine the subcellular localization of the protein. Therefore, we used vector pKD7-Red (Supplementary Figure 3A) to construct a recombinant vector (Supplementary Figure 3B), and the recombinant vector pKD7-Red-*CgPTPM1* was transformed into the deletion mutants using the ATMT method. Finally, the subcellular localization strains obtained were stained with MitoTracker^®^ Green FM (Invitrogen, Ltd., Paisley, United Kingdom), a dye specific to mitochondria. The co-localization of the RFP fluorescence signal and mitochondrial specific Mito-Green signal in hyphae and conidia observed with fluorescence inverted microscopy revealed that CgPTPM1 is primarily localized to the mitochondria of *C. graminicola* (Figure 2C). The results from this observation are also consistent with the previous predictive results (Table 1).

To determine that the changed phenotypes of the mutant were owing to the deletion of *CgPTPM1*, we obtained the complementation strain of *CgPTPM1*. In this study, since the subcellular localization strains were consistent with the complementation strains, we used one of the subcellular localization strains obtained as a complementation strain for the following phenotype tests and designated it Δ*CgPTPM1*/*CgPTPM1*.

### *CgPTPM1* Is Important for the Conidiation, Development, and Differentiation of *C. graminicola*

To study the biological functions of *CgPTPM1*, we constructed a recombinant vector targeting gene replacement and transformed it into the wild type M1.001 using the ATMT method. Finally, three gene knockout strains were obtained using the homology double exchange method (Supplementary Figure 2B).

**FIGURE 3:**
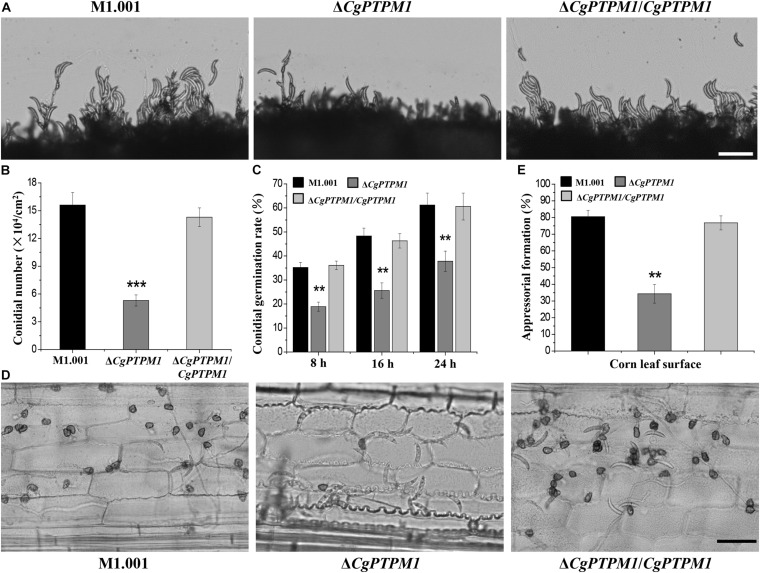
*CgPTPM1* is related to conidiation, conidial germination and the formation of appressoria. **(A)** Observation of conidiation. Conidia of wild type M1.001, the mutant Δ*CgPTPM1* and complementation strain Δ*CgPTPM1/CgPTPM1* from 14-day-old potato dextrose agar (PDA) media were transferred to glass slides and observed under an optical microscope. **(B)** Statistical analysis of the conidiation of strains cultured on PDA plates for 14 days. **(C)** Statistical analysis of conidial germination rates of strains on epidermal cells of corn leaves at 8, 16, and 24 h post infection (hpi). Conidial germination was measured at a concentration of 1 × 10^5^ conidia/mL, and more than 200 conidia were identified in each experiment. **(D)** Observation of the formation of appressoria on epidermal cells of corn leaves. A conidial suspension of 1 × 10^5^ conidia/mL was used to inoculate the corn leaf epidermal cells, and the formation of appressoria was observed at 24 hpi. **(E)** Statistical analysis of the rates of formation of appressoria of strains on corn leaf epidermal cells at 24 hpi. More than 200 appressoria in the experiment were identified. Error bars represent ± SD of three independent repeated samples. Two asterisks (**) represent an extremely significant difference at 0.001 < *P* < 0.01, and three asterisks (***) represent an extremely significant differences at *P* < 0.001. Scale bar = 50 μm.

When continuously cultured on PDA or CM plates for 7 days, it was observed that Δ*CgPTPM1* grew at a rate similar to that of the wild type and complementation strains based on their colony sizes (Supplementary Figures 5A,B). In addition, the conidial morphology did not changed among different types of strains (Supplementary Figure 5C). Therefore, we hypothesized that *CgPTPM1* is not involved in vegetative growth and conidial morphogenesis. The statistical results showed that the conidiation of the mutant (5.6 ± 0.2 × 10^4^) was significantly reduced and was approximately one third of those of the wild type (15.6 ± 0.3 × 10^4^) and complementation strains (14.3 ± 0.4 × 10^4^) (Figure 3B). In addition, the observation of the process of the conidial formation on PDA plates revealed that the conidiation of the wild type and complementation strains was also significantly higher than that of Δ*CgPTPM1* (Figure 3A). Based on these results, we hypothesized that *CgPTPM1* is involved in the formation of the conidia of *C. graminicola.*

The normal germination of conidia and the development of appressoria are important for many pathogenic fungi to infect their host (Bergstrom and Nicholson, 1999; Deising et al., 2000). In the conidial germination test on the surface of corn leaves, partial conidia of the wild type (35.2 ± 2.1%) and complementation strains (36.1 ± 1.8%) had begun to germinate at 8 h, while only a few conidia of the mutant (18.9 ± 1.9%) had germinated during the same period. During the following 16 h and 24 h, the germination rates of the wild type reached 48.3 ± 3.3% and 61.2 ± 5%, respectively, and those of the complementation strain were 46.3 ± 3% and 60.6 ± 5.6%, respectively, versus 25.6 ± 3.2% and 37.8 ± 4.2% for the mutant, respectively. The conidial germination rate had clearly decreased, and there was a delay in the germination process (Figure 3C). The test results of conidial germination on artificial hydrophobic films and onion epidermal cells were consistent with those on leaves (Supplementary Figure 6A). These results strongly suggest that *CgPTPM1* could be involved in the conidial germination process of *C. graminicola*. Moreover, the delayed conidial germination caused by the deletion of *CgPTPM1* could be related to the decreased pathogenicity of the mutant in subsequent experiment.

The rate (34.3 ± 4.6%) of appressorial formation of the mutant at 24 hpi on corn leaf cells was lower than that of the wild type (80.5 ± 3.8%) and complementation strains (76.8 ± 4.2%) (Figures 3D,E). The appressorial formation on artificial hydrophobic films and onion epidermal cells (Supplementary Figures 6B,C) was similar to that on corn leaves. Therefore, we hypothesized that *CgPTPM1* plays a role in the formation of appressoria of *C. graminicola*. Since appressoria are the main structures of the fungus that infect the host, the defect in appressorial formation may also be one of the important reasons for the decline in pathogenicity.

### The Δ*CgPTPM1* Strain Is Defective in Pathogenicity

**FIGURE 4:**
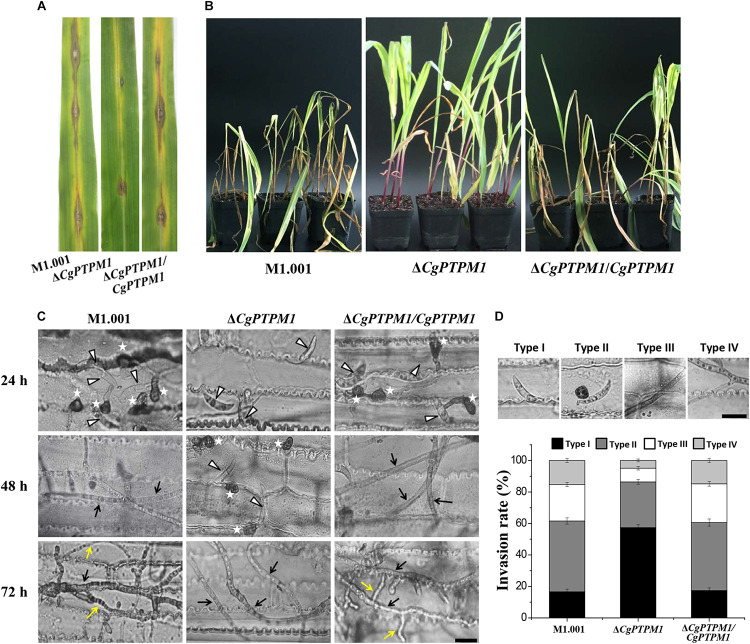
The absence of *CgPTPM1* reduces the pathogenicity of *C. graminicola*. **(A)** Leaf disease test. A conidial suspension of 10 μL of 5 × 10^5^ conidia/mL was inoculated on the third leaf of corn variety Xianyu 335, and the degree of infection was examined and photographed at 7 days post infection (dpi). **(B)** Whole plant disease test. Four week-old corn seedlings were sprayed with 5 mL of a conidia suspension with a concentration of 1 × 10^5^ conidia/mL. The results were examined at 7 days dpi and photographed. **(C)** Comparison of the infection structures formed by the wild type strain M1.001 and the mutant Δ*CgPTPM1* and complementation strains in different periods of infection. The appressoria are marked by asterisks. White triangles indicate conidia. Black arrows indicate primary hyphae, and yellow arrows indicate secondary hyphae. **(D)** Statistical analysis of each type of infection structure at 72 hpi. TypeI indicates ungerminated conidia. TypeII indicates the formation of appressoria. TypeIII indicates primary hyphae, and TypeIV indicates secondary hyphae. More than 200 infectious structures were counted for each strain investigated. The experiments were performed in triplicate and repeated three independent times for each strain. Error bars represent ± SD of three independently repeated samples. Scale bar = 10 μm.

The determination of pathogenicity revealed that the wild type and complementation strains could form typical lesion spots by placing 5 × 10^5^ conidia/mL suspensions on corn leaves after 7 days, and the lesions had combined into a large blotch on the leaf surface. Simultaneously, after inoculation with the mutant droplets, only a small lesion was formed on the leaf surface, and the degree of pathogenicity was significantly reduced (Figure 4A). Seven days after spraying 1 × 10^5^ conidia/mL conidial suspensions as inoculum on the whole corn plants, the wild type and complementation strains could cause severe disease on the whole corn in which the leaves were wilted and necrotic on a large scale. However, the pathogenicity of the mutant had clearly weakened, and only a small portion of the leaves were affected (Figure 4B). Based on these results, we concluded that *CgPTPM1* is involved in the pathogenic infection process of *C. graminicola*.

To further examine the role of *CgPTPM1* in the infection process, we conducted microscopic observations after spotting the leaves with the conidial suspension. The results showed that the wild type and complementation strains had begun to form appressoria at 24 hpi, while most of the mutant conidia were still in an ungerminated state. The wild type and complementation strains had formed primary hyphae at 48 hpi, while only a portion of the conidia of mutant began to germinate and form appressoria. The wild type and complementation strains had formed secondary hyphae on the basis of primary hyphae and grew as a necrotroph at 72 hpi, while the mutant had fewer invasive hyphae, and there was a delay in growth (Figure 4C). These results strongly suggest that *CgPTPM1* is involved in the infection process of *C. graminicola*, during which it participates in the pathogenic process by affecting the germination of conidia, the formation of appressoria and the expansion of hyphae. From the statistical results of infection types at 72 hpi (Figure 4D), the infection types of wild type and complementation strains were primarily concentrated on the appressoria (45.0 ± 2.0%, 43.2 ± 2.3%), primary hyphae (23.2 ± 1.3%, 24.6 ± 1.5%) and secondary hyphae (16.2 ± 1.3%, 15.8 ± 1.2%). However, during the same period, 57.4 ± 1.8% of the conidia of the mutant had not germinated, and the proportions of primary hyphae and secondary hyphae were only 8.7 ± 1.1% and 4.9 ± 0.9%, respectively. These results also provided evidence from microscopic observation for the role of *CgPTPM1* in pathogenesis.

To clarify the functions of *CgPTPM1* in more detail, we determined the relative expression of *CgPTPM1* in different periods of *C. graminicola* using qRT-PCR. The results (Supplementary Figure 7) indicated that the expression of *CgPTPM1* increased overall from the vegetative growth to pathogenic period. The level of expression of *CgPTPM1* was the highest at 48 hpi, which was approximately 10 times that of the period of vegetative hyphae. These results indicate that *CgPTPM1* may be involved in the physiological process of *C. graminicola* at various stages, but it primarily plays a role in the pathogenic process.

### *CgPTPM1* Is Associated With the Regulation of Excessive H_2_O_2_

**FIGURE 5:**
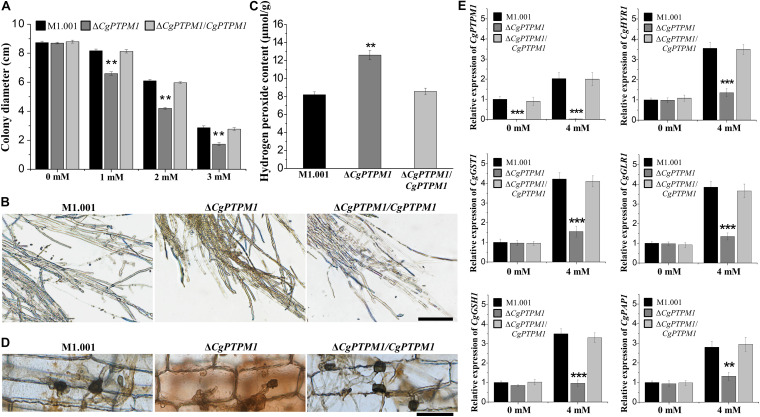
The mutant Δ*CgPTPM1* is more sensitive to H_2_O_2_ and cannot scavenge excessive H_2_O_2_ in a timely manner. **(A)** Statistical analysis of the colony diameters of wild type, mutant and complementation strains on complete minimal media (CM) plates that contain H_2_O_2_. **(B)** The results of 3,3′-diaminobenzidine (DAB) staining of vegetative hyphae after three types of strains were cultured on CM plates for 4 days. **(C)** Determination of the concentration of H_2_O_2_ in the wild type, mutant and complementation strains. **(D)** DAB staining of the excised leaves of corn infected by the wild type, Δ*CgPTPM1* and complementation strains at 48 h post infection. **(E)** The expression of *CgPTPM1* could affect the expression of antioxidant gene orthologs in *C. graminicola*. Compared with the 0 mM H_2_O_2_ treatment, the expression of *CgPTPM1* in wild type and complementation strains increased when induced with 4 mM H_2_O_2_, and the expression of the other five antioxidant-related genes *CgHYR1*, *CgGST1*, *CgGLR1*, *CgGSH1* and *CgPAP1* was upregulated. In the mutant, *CgPTPM1* was not expressed, and the expression of the other five genes did not change significantly. Two asterisks (**) represent an extremely significant difference at 0.001 < *P* < 0.01, and three asterisks (***) represent an extremely significant differences at *P* < 0.001. Error bars represent ± SD of three independent repeated samples. Scale bar = 20 μm.

After invading the host cell, pathogenic fungi should respond to the ROS defense response produced by the host cell (Apostol et al., 1989; Doke et al., 1996; Mellersh et al., 2002). To study the role of *CgPTPM1* in response to exogenous ROS, strains were cultured in the dark for 7 days on CM plates that contained different concentrations of H_2_O_2_. The results showed that the mutant was more sensitive to exogenous H_2_O_2_ as the concentrations increased (Figure 5A; Supplementary Figure S8A). Therefore, we preliminarily hypothesized that *CgPTPM1* plays a role in the process of pathogens by responding to exogenous H_2_O_2_. The timely removal of the ROS produced by the host is an important way for pathogenic fungi to respond to plant immune defense, and it can also promote their infection process (Chi et al., 2009; Guo et al., 2010). Based on these results, we hypothesized that the reduced pathogenicity of Δ*CgPTPM1* may be related to its inability to clear the exogenous ROS produced by the host in a timely manner. Therefore, we used DAB staining to detect the removal of ROS in corn leaf cells at 48 hpi. The results showed that there was no accumulation of brown product in the cells infected by the wild type and complementation strains, while the cells infected by Δ*CgPTPM1* had a brown product around the hyphae and appressoria (Figure 5D). This result provided additional evidence indicating that *CgPTPM1* may be involved in the process of the pathogen response to exogenous H_2_O_2_, specifically by removing the H_2_O_2_ produced by the host during the infection process.

An appropriate amount of H_2_O_2_ can be used as a signaling molecule to participate in physiological regulation in the organism, but excessive H_2_O_2_ can cause damage to cells (Egan et al., 2007; Yang et al., 2015). To study the relationship between *CgPTPM1* and endogenous H_2_O_2_, we stained with DAB and determined the levels of endogenous H_2_O_2_ on the vegetative hyphae of wild type and mutant and complementation strains. The brown product accumulated to a lesser extent in the wild type and complementation strains, while a greater amount of brown product formed in the mutant (Figure 5B). Moreover, we also found that the content of endogenous H_2_O_2_ in the mutant was approximately 1.5 times that of the wild type and complementation strains (Figure 5C). The results also indicated that the deletion of *CgPTPM1* may cause an inability to scavenge the excess H_2_O_2_ in a timely manner. Combined with the previous results of *CgPTPM1* in the process of managing exogenous H_2_O_2_, we hypothesized that *CgPTPM1* is related to the regulation of excess H_2_O_2_.

It has been reported that mitochondria play an important role in the production of ROS in most organisms (Friguet, 2006; Zhang et al., 2011). Through the previous subcellular localization results, we also learned that CgPTPM1 is primarily localized to mitochondria. Therefore, we hypothesized that the process of the regulation of excess H_2_O_2_ by *CgPTPM1* may be achieved by regulating certain genes related to the scavenging of H_2_O_2_. To verify this hypothesis, we determined the effect of the expression of *CgPTPM1* following induction by H_2_O_2_ on the levels of expression of the other five genes *CgHYR1*, *CgGST1*, *CgGLR1*, *CgGSH1* and *CgPAP1*, which are related to the scavenging of H_2_O_2_. The results showed that following induction with 4 mM H_2_O_2_, the levels of expression of *CgPTPM1* in the wild type and complementation strains had increased significantly compared with the absence of H_2_O_2_ induction, while the levels of expression of the other five genes were also upregulated to varying degrees. However, the expression of *CgPTPM1* was not detected in the mutant, while the levels of expression of the other five genes did not change significantly even following induction by 4 mM H_2_O_2_ (Figure 5E). Therefore, we hypothesized that *CgPTPM1* participates in the regulation of excessive H_2_O_2_ by regulating a series of genes related to H_2_O_2_ degradation, and these regulatory processes may involve a variety of signal transduction pathways.

## Discussion

Approximately one-third of the proteins in eukaryotic cells can be phosphorylated, and the reversible phosphorylation process is simultaneously regulated by protein kinases and PPs. This process is related to many physiological processes, such as vegetative growth, cell division and signal transduction (Zolnierowicz and Bollen, 2000; Cohen, 2001; Tonks, 2013). Corn anthracnose is a serious fungal disease worldwide, causing extremely high annual losses in the production of corn (Dean et al., 2012; O’Connell et al., 2012). In this study, we identified a putative tyrosine phosphatase protein CgPTPM1 in the pathogenic fungus *C. graminicola* that causes corn anthracnose. The phosphatase domains and catalytic motifs in the homologs of PTPs are also highly conserved among various species (Supplementary Figure 1). The determination of its enzymatic activity showed that CgPTPM1 has phosphatase activity (Figure 2B), and analysis of subcellular localization indicated that the protein is primarily distributed on the mitochondria (Figure 2C).

Some studies had described that PTPs will affect the vegetative growth of filamentous pathogenic fungi. For example, the deletion of putative PTP gene *MoPTP2* in *M. oryzae* could cause a restriction in the growth of pathogenic fungus (Bohnert et al., 2019). In addition, the genes *BcPTPA* and *BcPTPB* that encode the putative PTPs in *Botrytis cinerea* also affected the normal growth of the fungus (Yang et al., 2013). However, in this study, the lack of *CgPTPM1* did not affect the growth of the mutant Δ*CgPTPM1*. In another pathogenic fungus, *Fusarium graminearum*, knockout of the putative PTP gene *FgTEP1* also did not affect the vegetative growth of the pathogen (Zhang et al., 2010). The reason for this difference could be that the PTPs have formed different types during the process of evolution, and there are also differences in functions of each type. For example, MoPTP2 in *M. oryzae* and BcPTPA in *B. cinerea* are homologs of PTP ScPTP2 in *S. cerevisiae*, and BcPTPB is a homolog of ScPTP3. Alternatively, FgTEP1 of *F. graminearum* is closely related to *CgPTPM1* in this study (Figure 1C). Therefore, the difference in classification also leads to different functions of various types of phosphatases.

The generation of falcate conidia and their dispersal with rain and wind are the main ways that *C. graminicola* spreads and causes infections in the field (Bergstrom and Nicholson, 1999; Wang et al., 2016). In this study, the conidial morphology of the mutant did not change compared with that of the wild type, but the conidiation decreased by approximately 66% (Figures 3A,B). Therefore, this suggests that the *CgPTPM1* gene may be involved in the regulatory process of conidial formation. The effect of PTPs on fungal sporulation has been studied. For example, Heymont et al. (2000) showed that the phosphatase gene *ScTEP1* reduces the ability of *S. cerevisiae* to sporulate by affecting its process of meiosis, which is accompanied by a decrease in the accumulation of dityrosine, a major component of the yeast cell wall. In addition, the deletion of the related PTPs genes *FgTEP1* in *F. graminearum* or *UmPtn1* in *U. maydis* also affect the formation of spores in these pathogenic fungi (Zhang et al., 2010; Vijayakrishnapillai et al., 2018). Although we still do not know the specific reasons for the decrease in conidial production caused by the deletion of *CgPTPM1*, based on the results of previous studies, we hypothesized that this gene may be involved in some signaling pathways that result in the reduction of conidiation. Simultaneously, some studies have shown that spore germination and appressorial formation represent the start of most filamentous fungal infections (Mims and Vaillancourt, 2002; Wang et al., 2016). These processes are regulated by many signaling pathways, such as the cAMP/PKA signaling pathway, MAPK signaling pathway and Ca^2+^-mediated signaling pathway (Adachi and Hamer, 1998; Li et al., 2012; Marroquin-Guzman and Wilson, 2015; Jiang et al., 2018). With the contact between conidia and the host, physical signals of the plant surface, such as tissue hardness and hydrophobicity, as well as chemical signals from leaf wax and keratin-derived fatty acids, can induce spore germination and appressorial formation (Deising et al., 2000; Li et al., 2012; Marroquin-Guzman and Wilson, 2015). The appressorium is the most important specialized cell in the pathogenic process of *C. graminicola*. Whether it can develop normally and successfully invade host cells is crucial for pathogens, and this process is also regulated by various signals (Marroquin-Guzman and Wilson, 2015; Yan et al., 2018). Liu et al. (2016) have shown that knocking out the putative DUSP gene *MoYVH1* greatly changed the vegetative growth, spore production, pathogenic ability and sensitivity to ROS of *M. oryzae*, and this process is achieved through the interaction of MoYvh1 with the ribosome maturation factor MoMrt4 (Liu et al., 2018). Qian et al. (2018) showed that the serine/threonine phosphatase MoPpe1 and its functionally similar MoSap1 interact with MoMkk1 as a complex participant in the MAPK signaling pathway and regulate CWI and target of rapamycin (TOR) pathway of *M. oryzae*, which caused a decline in the functions of appressoria and attenuated the pathogenicity of the pathogen. In this study, the conidial germination rate and rate of appressorial formation of Δ*CgPTPM1* were reduced to varying degrees compared with the wild type (Figures 3C–E; Supplementary Figure 6), and there was a delay in the germination of conidia. Based on these conclusions, we hypothesized that there might be some intermediate receptors that can interact with CgPTPM1 during physiological functions, thereby regulating the factors of action of certain signaling pathways. In addition, CgPTPM1 may act as a final receptor affecting the kinase during the process of phosphorylation homeostasis by dephosphorylation to regulate the processes of spore formation, germination and the maturation of appressoria.

The ROS produced by NADPH oxidases on the plasma membrane of host cells are considered to be some of the compounds that are rapidly produced in the defense response of plants to resist the invasion of pathogens (Apostol et al., 1989; Doke et al., 1996; Mellersh et al., 2002; Belozerskaya and Gessler, 2007). Whether it can effectively release the toxicity of exogenous ROS is also the key to whether the pathogen can successfully infect the host. Some research has shown that many genes related to ROS in pathogenic fungi also affect their own pathogenicity. For example, in *M. oryzae*, *MoHYR1*, *MoAP1*, *MoTIG1*, and *MoMAP1* can regulate the oxidative stress process, toxicity and pathogenicity of this fungus (Ding et al., 2010; Guo et al., 2011; Huang et al., 2011; Li et al., 2015). In this study, the mutant had a stronger sensitivity to high concentrations of exogenous H_2_O_2_ compared with the wild type (Figure 5A; Supplementary Figure 8A). In the DAB staining assay, after the corn leaves were inoculated, it was also observed that the leaf cells infected by the mutant accumulated more of the brown product (Figure 5D). We hypothesized that the reason could be that the mutant could not scavenge the high concentration of H_2_O_2_ produced by the host in a timely manner. Interestingly, when we dyed the vegetative mycelia of different types of strains with DAB, we found that the mycelia of mutant also accumulated a darker brown substance (Figure 5B), and quantitative measurements revealed that the level of endogenous H_2_O_2_ in the mycelia of mutant was also higher than that of the wild type and complementation strains cultured in the same manner (Figure 5C). This result proved that with the deletion of *CgPTPM1*, in addition to exogenous H_2_O_2_, *C. graminicola* also cannot scavenge the endogenous H_2_O_2_ produced by itself in a timely manner. The subcellular localization analysis showed that CgPTPM1 is primarily located to the mitochondria of *C. graminicola*. Mitochondria, as functional organelles, also participate in the production of ROS in the organism. Simultaneously, studies in *M. oryzae* indicated that glutathione peroxidase MoHyr1 acts on MoYap1 to remove ROS in host cells, and when the expression of *MoHYR1* increases under the induction of H_2_O_2_, the levels of expression of a series of genes related to ROS clearance are upregulated (Huang et al., 2011). In this study, we also found that the expression of the *CgPTPM1* gene in the wild type caused an upregulation of the levels of expression of predicted genes related to ROS scavenging (Figure 5E). Therefore, we hypothesized that *CgPTPM1* may scavenge excess H_2_O_2_ by acting on the genes related to ROS clearance in *C. graminicola*. In addition, osmotic regulation plays a role in the response of many pathogenic fungi to oxidative stress, and mutants with blocked osmotic pathways become more sensitive to active oxidation (Hamel et al., 2012; Li et al., 2012). However, in this study, the mutants were not found to be sensitive to high concentrations of NaCl and sorbitol (Supplementary Figure 9). Therefore, it is hypothesized that the *CgPTPM1* gene may be relatively independent between the regulation of oxidative stress and the osmoregulatory pathway in *C. graminicola*.

Studies have shown that the MAPK signaling pathway related to CWI is relatively conservative in the pathogenic process of fungal pathogens (Zhao et al., 2007), and this pathway has been studied in many fungi. For example, in *S. cerevisiae*, Slt2 regulates the CWI and promotes the synthesis of the cell wall (Heinisch, 2005). Although the homolog Mps1 of Slt2 in the hemibiotrophic ascomycete *M. oryzae* does not affect the formation of appressoria, it is essential for the CWI, the infection of appressoria and hyphal growth after infection (Xu et al., 1998; Rebollar and Lopez-Garcia, 2013). Yang et al. (2013) had also delineated that the putative PTP genes *BcPTPA* and *BcPTPB* in the necrotrophic pathogen *B. cinerea* affect the integrity of cell wall and pathogenicity through the CWI signaling pathway. In this study, the sensitivity of the mutant to CR and SDS, which affect the synthesis of the cell wall, was enhanced, and the rate of colony growth on the CM plates that contained CR and SDS was significantly lower than those of the wild type and complementation strains (Supplementary Figure 9). There is some intersection between various signaling pathways in pathogenic fungi (Li et al., 2012). For example, in *M. oryzae*, the gene encoding putative DUSP MoYvh1 as a common upstream element of the cAMP-PKA and CWI MAPK pathways that regulate physiological functions, such as CWI and pathogenicity (Liu et al., 2016). In addition, the MoPpe1-MoSap1 complex mediates the cross-response process between CWI and the TOR signaling pathways in *M. oryzae* (Qian et al., 2018). Therefore, we hypothesized that CgPTPM1 may act as a regulatory factor for the CWI MAPK signaling pathway while participating in other physiological activities of the pathogen, thereby affecting the integrity of the cell wall.

The effects of functional genes on the pathogenicity of pathogenic fungi are always critical aspects of study for researchers. In this study, the pathogenicity of Δ*CgPTPM1* was clearly reduced compared with the wild type (Figures 4A,B), and this result could be caused by several reasons. First, the conidiation of the mutant decreased (Figures 3A,B; Supplementary Figure 5C). The conidia play an important role in the survival and development of the pathogen in the field; moreover, the falcate conidia are the main transmission mode of *C. graminicola* (Bergstrom and Nicholson, 1999; Wang et al., 2016). Therefore, the decrease in conidia could directly affect the pathogenicity of the fungus. Secondly, owing to the germination of conidia, the development and maturation of appressoria and the normal growth of hyphae are all related to the pathogenic process (Deising et al., 2000; Werner et al., 2007; Ludwig et al., 2014). Therefore, the defects of the mutants in these aspects may also be a reason for the weakened pathogenic ability. Third, clearance of the exogenous H_2_O_2_ produced by the host in a timely manner and the maintenance of endogenous H_2_O_2_ level in the strains is very critical for pathogens. In this study, the sensitivity of the mutant to high concentrations of H_2_O_2_ (Figure 5A; Supplementary Figure 8A) and the inability to scavenge the excessive H_2_O_2_ produced by the host (Figure 5D) and itself (Figures 5B,C) may also have led to the weakening of its pathogenic ability. In addition, the effect of *CgPTPM1* on the expression of genes related to H_2_O_2_ scavenging may also be one of the reasons for the decreased pathogenicity of the mutant. Finally, with the deletion of *CgPTPM1*, the CWI of *C. graminicola* was destroyed (Supplementary Figure 9). Previous studies have shown that the CWI signaling pathway plays an important role in the development and pathogenicity of plant pathogenic fungi (Li et al., 2012; Jiang et al., 2018). Therefore, we hypothesized that there is some relationship between the reduction in pathogenicity and the damage to integrity of the cell wall. In summary, there are many reasons for the reduction of the pathogenicity of the Δ*CgPTPM1*, and the effect of *CgPTPM1* on the regulation of pathogenicity may be achieved by its participation in multiple signaling pathways.

Although protein phosphorylation is regulated by both kinases and phosphatases, the current research focus is primarily on kinases. To our knowledge, there are currently very few reports on the effect of phosphatase in *C. graminicola*. Therefore, the study of phosphatase is helpful to fully understand the role of phosphorylation in the physiology and pathogenic process of this pathogen. Our results showed that compared with the wild type strain, the mutant produced fewer conidia; the conidial germination rate was reduced, and the formation of appressoria and the extension of infection hyphae were inhibited. In addition, *CgPTPM1* is also involved in the process of the pathogen response to excessive ROS and the regulation of CWI. Moreover, the reduced pathogenicity of the mutant is also related to the role of *CgPTPM1* in the physiological processes decreased. In summary, the results of this research introduce new views in the study of phosphatase in *C. graminicola*, add theoretical support for the development of modern agriculture, and provide some reference for the design of a drug to target the gene. Therefore, it has important scientific significance and practical value.

## Data Availability Statement

The raw data supporting the conclusions of this article will be made available by the authors, without undue reservation.

## Author Contributions

S-HZ and GL designed the research. SW performed the research. YD and PZ assisted in part of the experimental process. SW, GL, and S-HZ analyzed the data. S-HZ and SW wrote the manuscript. All authors contributed to the article and approved the submitted version.

## Conflict of Interest

The authors declare that the research was conducted in the absence of any commercial or financial relationships that could be construed as a potential conflict of interest.

## Footnotes

1 http://www.ncbi.nlm.nih.gov

2 http://fungi.ensembl.org/

3 http://www.softberry.com/berry.phtml

4 https://ihg.gsf.de/ihg/mitoprot.html

5 http://smart.embl-heidelberg.de/

6 https://www.megasoftware.net/

7 https://swissmodel.expasy.org/

8 https://web.expasy.org/compute_pi/

## Abbreviations

ATMT: *A. tumefaciens*-mediated transformation
CM: complete minimal media
CR: Congo red
CWI: cell wall integrity
DAB: 3,3 ′ -diaminobenzidine
DUSP: dual-specificity phosphatase
hpi: hours post inoculation
MAPK: mitogen-activated protein kinase
PDA: potato dextrose agar
pNPP: *p*-nitrophenyl phosphate
PP: protein phosphatase
PTP: protein tyrosine phosphatase
qRT-PCR: quantitative real-time PCR
ROS: reactive oxygen species
TOR: target of rapamycin.
