# Supplementary material for: Involvement of the Mitochondrial Protein Tyrosine Phosphatase PTPM1 in the Promotion of Conidiation, Development, and Pathogenicity in Colletotrichum graminicola
Source: Front Microbiol. 2021 Jan 14;11:605738. doi: 10.3389/fmicb.2020.605738 (PMC7841309; doi:10.3389/fmicb.2020.605738)
Supplement: Supplementary file 1 [file Data_Sheet_1.docx]

***Supplementary Material***

**Figure S1**. Full-length sequence alignment of the CgPTPM1 with homologs reported in other species. The dark blue indicates that the homology of sequences is 100%. The pink indicates homology ≥75%, and the cyan indicates homology ≥50%.

**Figure S2**. Construction and verification of *CgPTPM1* deletion mutants. **(A)** Structure of the knockout vector pXEH 2.0. **(B)** The knockout strategy of *CgPTPM1* gene. G-L: upstream flanking sequence of *CgPTPM1*. G-R: downstream flanking sequence of *CgPTPM1*. The genomic DNA of the M1.001 strain was amplified for upstream and downstream flanking sequences of *CgPTPM1*, which was cloned between the *Eco*RⅠ-*Kpn*Ⅰ and *Xba*Ⅰ-*Sal*Ⅰ sites of the PXEH 2.0 vector to generate the replacement vector. HPH: hygromycin B phosphotransferase resistance gene. **(C)** The *HPH* gene (Lane 1-4), upstream fragment of *CgPTPM1* and a partial *HPH* fusion gene (Lane 5-8) and target gene (Lane 9-12) were amplified with genomic DNA extracted from deletion strains No.1, No. 4, No. 8 and wild type as templates. M: DNA markers (2000 bp, 1000 bp, 750 bp, 500 bp, 250 bp and 100 bp). **(D)** Detection of the levels of expression of deletion strains (No.1, No. 4 and No. 8) versus the wild type M1.001 by qRT-PCR.

**Figure S3**. Acquisition and verification of the subcellular localization strain. **(A)** Structure of vector pKD7-Red. PH3: H3 promoter. DsRed2: red fluorescence gene. NEO: neomycin phosphotransferaseⅡ gene. RP27 ter: terminator. **(B)** Strategy of the construction of recombinant vector. The ORF fragment without an intron and the stop codon of *CgPTPM1* was amplified from M1.001 genomic cDNA and cloned between the *Sma*Ⅰ sites of the pKD7-Red vector to generate the final fusion vector. **(C)** Verification of the subcellular localization strain. **(a)** Verification of the fusion gene by PCR. M: DNA marker. Lane 1: fusion gene. Lane 2: negative control. **(b)** Detection of the level of expression of the localization strain versus the wild type M1.001 by qRT-PCR.

**Figure S4**. Acquisition and verification of the complementation strain. **(A)** Structure of vector pCB1532. **(B)** Strategy of the construction of recombinant vector. The coding sequence of *CgPTPM1* was amplified from M1.001 genomic DNA and cloned between the *Hind*Ⅲ and *Kpn*Ⅰsites of the pCB1532 vector to generate the recombinant vector. **(C)** Verification of the complementation strain. **(a)** Verification of the target gene by PCR. M: DNA marker. Lane 1: negative control. Lane 2 and 3: The target genes of complementation strains 1 and 2. **(b)** Detection of the levels of expression of the complementation strains versus the wild type M1.001 by qRT-PCR.

**Figure S5**. Vegetative growth, conidial morphology and conidiation. **(A)** Wild type, mutant and complementation strains were cultured on potato dextrose agar (PDA) or complete minimal media (CM) plates at 25°C for 7 days. The growth of the colonies was observed and photographed. **(B)** Statistical analysis of the colony diameters of three types of strains. Error bars represent ± SD of three independent repeated samples. **(C)** Observation of conidial morphology and conidiation. The strains were cultured on PDA plates under continuous white lighting for 14 days to induce the production of conidia. The conidia were harvested using 10 mL of sterile water, and then a conidial suspension was prepared. The morphology of a 10 µL sample was observed and counted using an optical microscope. Scale bar = 50 μm.

**Figure S6**. Conidial germination and appressorial formation on artificial hydrophobic films and onion epidermal cells. **(A)** Statistics of the rate of conidial germination. **(B)** Observation of appressorial formation on artificial hydrophobic films **(a)** and onion epidermis **(b)**. **(C)** Statistics of the rate of appressorial formation. More than 200 conidia were counted for each strain investigated. Error bars represent ± SD of three independently repeated samples. Two asterisks (**) represent extremely significant differences between the data at P < 0.01. Scale bar = 50 μm.

**Figure S7**. The relative levels of expression of samples of *C. graminicola* in different periods were determined using qRT-PCR. The actin gene GLRG_03056 was used as a reference for normalization. The levels of expression of messenger RNA were calculated using the 2^-ΔΔCt^ method. Error bars represent ± SD of three independently repeated samples.

**Figure S8**. The mutant displays greater sensitivity to H_2_O_2_. **(A)** Growth of the wild type M1.001, Δ*CgPTPM1* and complementation strains after being cultured on CM plates containing 0 mM, 1 mM, 2 mM and 3 mM of H_2_O_2_ for 7 days. **(B)** The H_2_O_2_ standard curve was determined by the addition of a complex of 0-5 μM titanium-H_2_O_2_ into seven 15 mL centrifuge tubes. The tubes were centrifuged at 3000 g for 10 min, and the supernatant was discarded. Two molar sulfuric acid was used to fully dissolve the precipitate, which was then diluted to 6 mL. The absorbance of each tube was measured at 415 nm.

**Figure S9**. Detection of osmotic stress and CWI. **(A)** Wild type M1.001, Δ*CgPTPM1* and complementation strains were cultured on CM plates that contained 2.5％ and 5％ (w/v) NaCl or sorbitol, 0.1 mg/mL and 0.2 mg/mL CR, or 0.005％ and 0.01％ (w/v) SDS for 7 days. The colony diameters were measured and photographed. **(B)** Statistical analysis of colony growth. The results showed that *CgPTPM1* is not involved in the response of pathogen to exogenous osmotic stress but affects the cell wall integrity. Error bars represent ± SD of three independent repeated samples. The two asterisks (**) represent extremely significant differences between the data at P < 0.01.

**Table S1. Primers used in research**

| Primer Name | Sequence 5′-3′ |
| --- | --- |
| GLRG-qc-LF | CGGAATTCCTGTCGGCAACCAGGGAAC |
| GLRG-qc-LR | GGGGTACCGTGGAAGCGGAGCAAACGT |
| GLRG-qc-RF | GCTCTAGATCTTTTTCCATCTCCACGG |
| GLRG-qc-RR | GCGTCGACTGACTATTCTCACGCACGC |
| CgPTPM1-F | ACCTTTGCTATGTCACTTCCG |
| CgPTPM1-R | GCTTGTCTTCGACCACTTTCT |
| HYG-YZ-F | GCCCTTCCTCCCTTTATTTC |
| HYG-YZ-R | ACTCTATTCCTTTGCCCTCG |
| G-L-F | GGTTCGCCTCTTTCTCTCC |
| G-L-R | ACTCTATTCCTTTGCCCTCG |
| GLRG-BD-F | CCAAGCTTGCATGGCTTCTCTGTTGCGA |
| GLRG-BD-R | CCCTCGAGCTTCCTCACCTCTGTGGTCC |
| CgPTPM1HB-F | TCCCCCGGGATGGCTTCTCTGTTGCGA |
| CgPTPM1HB-R | TCCCCCGGGCTTCCTCACCTCTGTGGT |
| GLRG-HB-Red-F | CCGACTGGAAGGGTGGCAACA |
| GLRG-HB-Red-R | GCCGTACTGGAACTGGGGGGA |
| G-hb-F | CCCAAGCTTCTCTTTCTCTCCGCCATTAC |
| G-hb-R | GGGGTACCCTTTCGGTTAGGCTCTTAGC |
| Gq-YP-F | GTGTGGAACATAGCGCAAAC |
| Gq-YP-R | TTCGGCTTGAGGATAACAGC |
| Gq-actin-F | GATTTGGCACCACACTTTC |
| Gq-actin-R | TCTTCTCTCTGTTGGACTT |
| HYR1-qPCR-F | TCTGCCAGGTCAATTACGGC |
| HYR1-qPCR-R | CTGGAAGAGAGGGTTTGCGT |
| GST1-qPCR-F | GTCAAGGGCAACGAACAACG |
| GST1-qPCR-R | ATACGGTGTCTCCGGAAGGA |
| GLR1-qPCR-F | CCGAAGAGAAGGTTGTCGGT |
| GLR1-qPCR-R | GGTGGATGGCAACACAACTG |
| GSH1-qPCR-F | TAAGGGCTTCCTCGCAAACA |
| GSH1-qPCR-R | TAGGTCGAGTTGGAGGCGTA |
| PAP1-qPCR-F | GAGCTGTCCTAGTGTGCAGG |
| PAP1-qPCR-R | CCGTCTCAGAAACAACCGGA |

Note: Sequences which marked with “___” stand for restriction enzyme cutting site

**Table S2. Reagent formula of the H_2_O_2_ standard curve**

| Reagent (mL) | Centrifuge tube number | | | | | | |
| --- | --- | --- | --- | --- | --- | --- | --- |
|  | 1 | 2 | 3 | 4 | 5 | 6 | 7 |
| 5mM H_2_O_2_ | 0 | 0.1 | 0.2 | 0.3 | 0.6 | 0.8 | 1.0 |
| Cold Acetone | 1.0 | 0.9 | 0.8 | 0.6 | 0.4 | 0.2 | 0 |
| 5％ Titanic Sulfate | 0.1 | 0.1 | 0.1 | 0.1 | 0.1 | 0.1 | 0.1 |
| Strong Aqueous Ammonia | 0.2 | 0.2 | 0.2 | 0.2 | 0.2 | 0.2 | 0.2 |
