# Supplementary figures and images for: Involvement of the Mitochondrial Protein Tyrosine Phosphatase PTPM1 in the Promotion of Conidiation, Development, and Pathogenicity in Colletotrichum graminicola
Source: Front Microbiol. 2021 Jan 14;11:605738. doi: 10.3389/fmicb.2020.605738 (PMC7841309; doi:10.3389/fmicb.2020.605738)

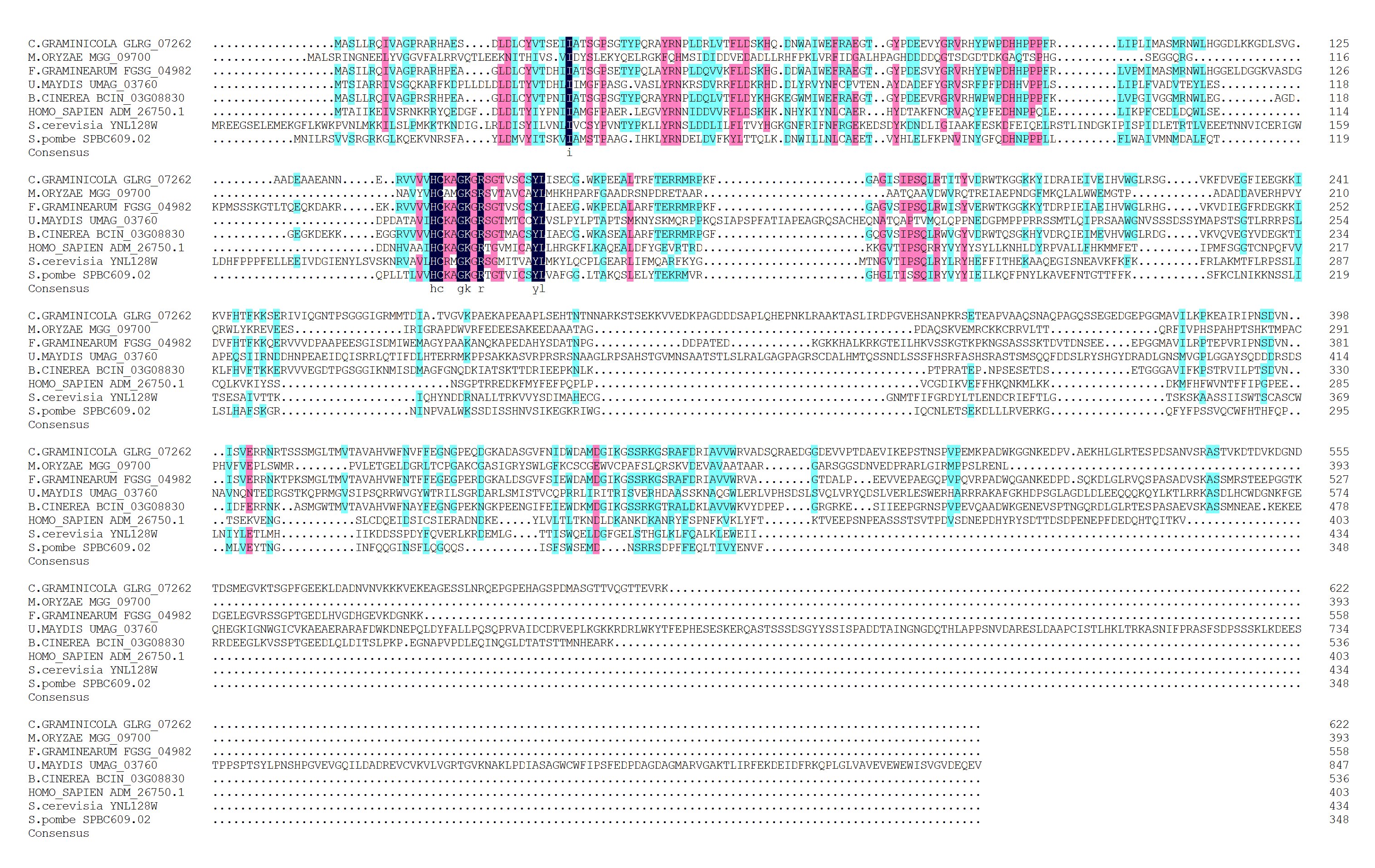

Supplement: Supplementary file 2 [file Image_1.TIF]

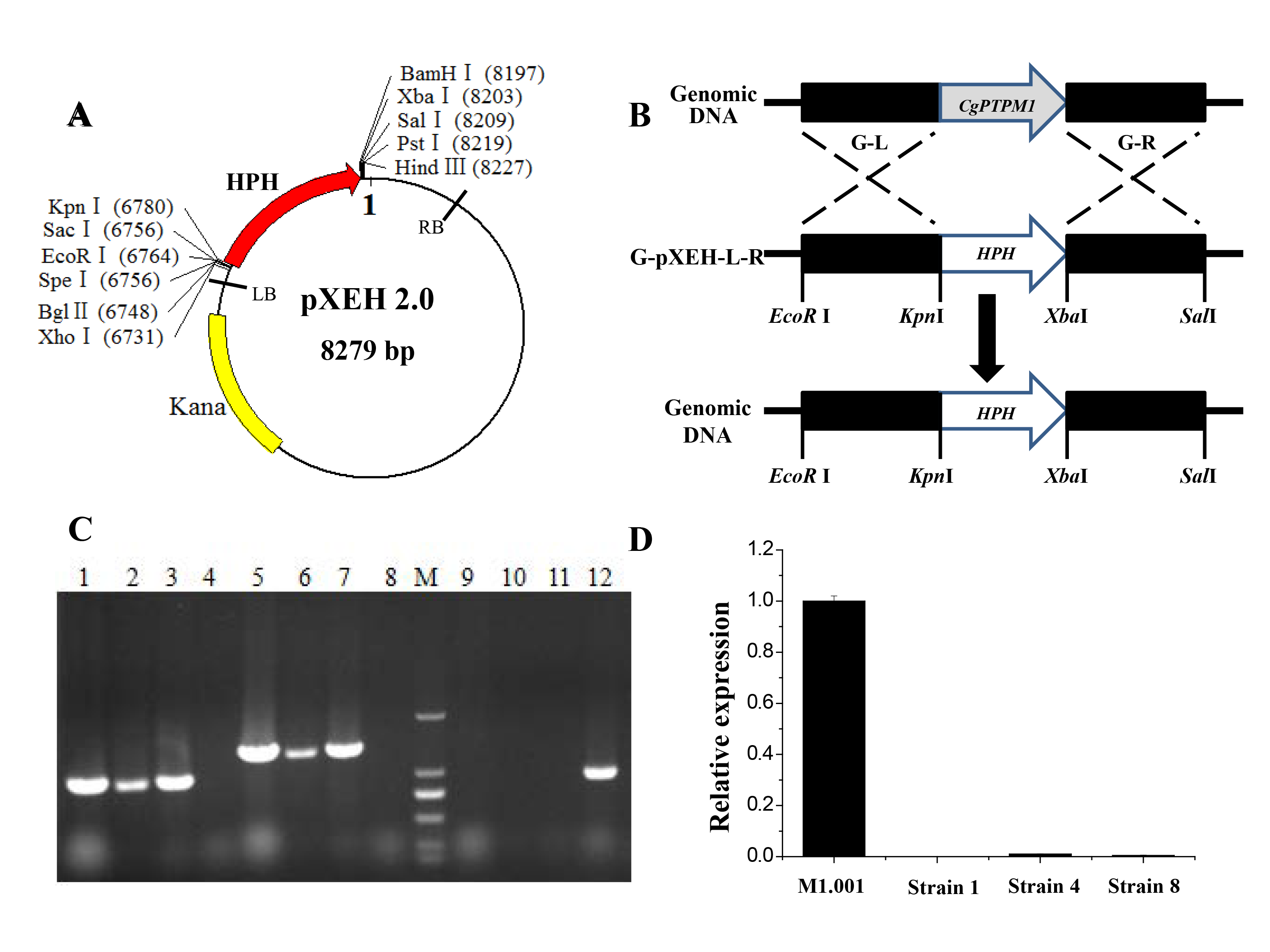

Supplement: Supplementary file 3 [file Image_2.TIF]

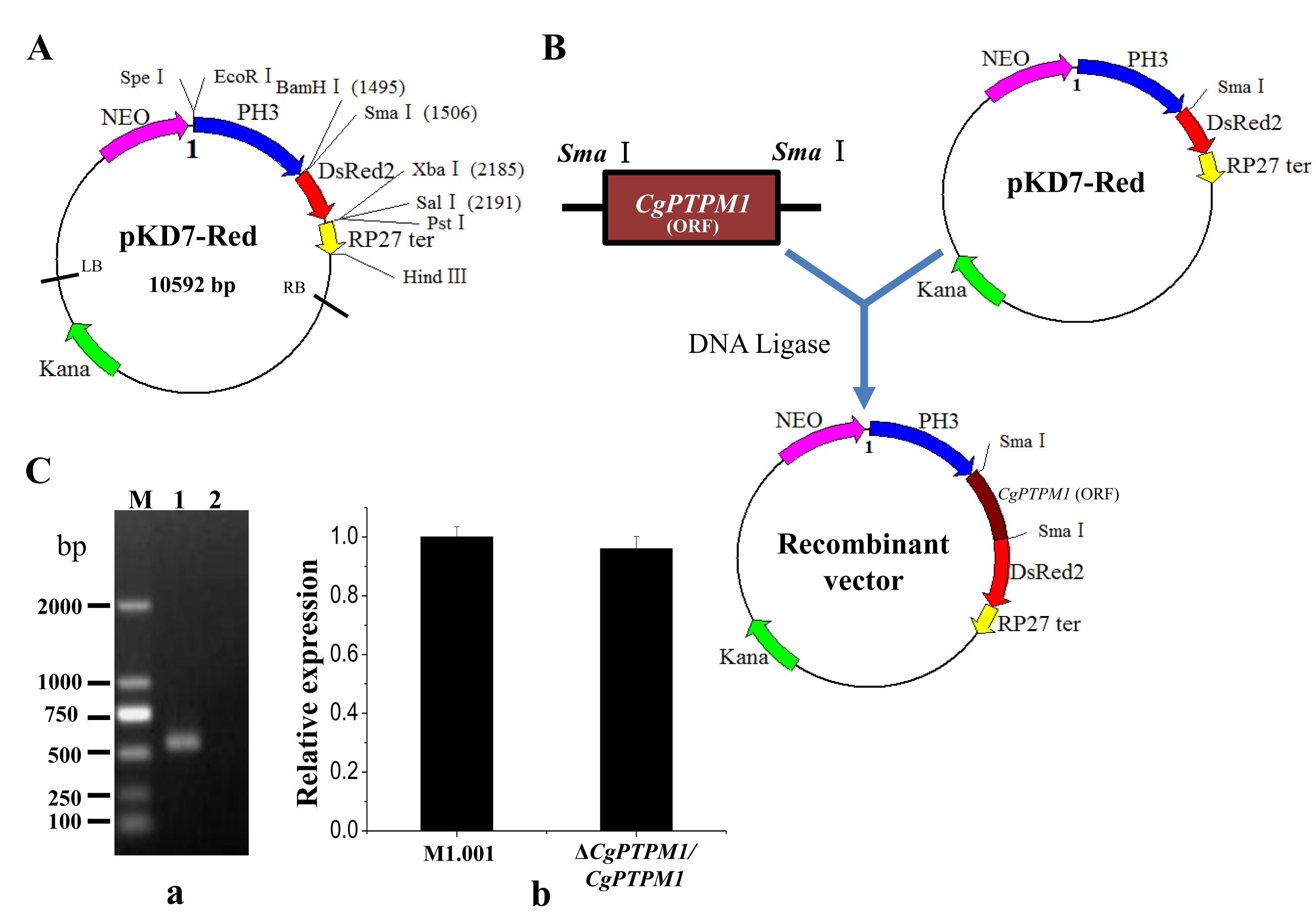

Supplement: Supplementary file 4 [file Image_3.TIF]

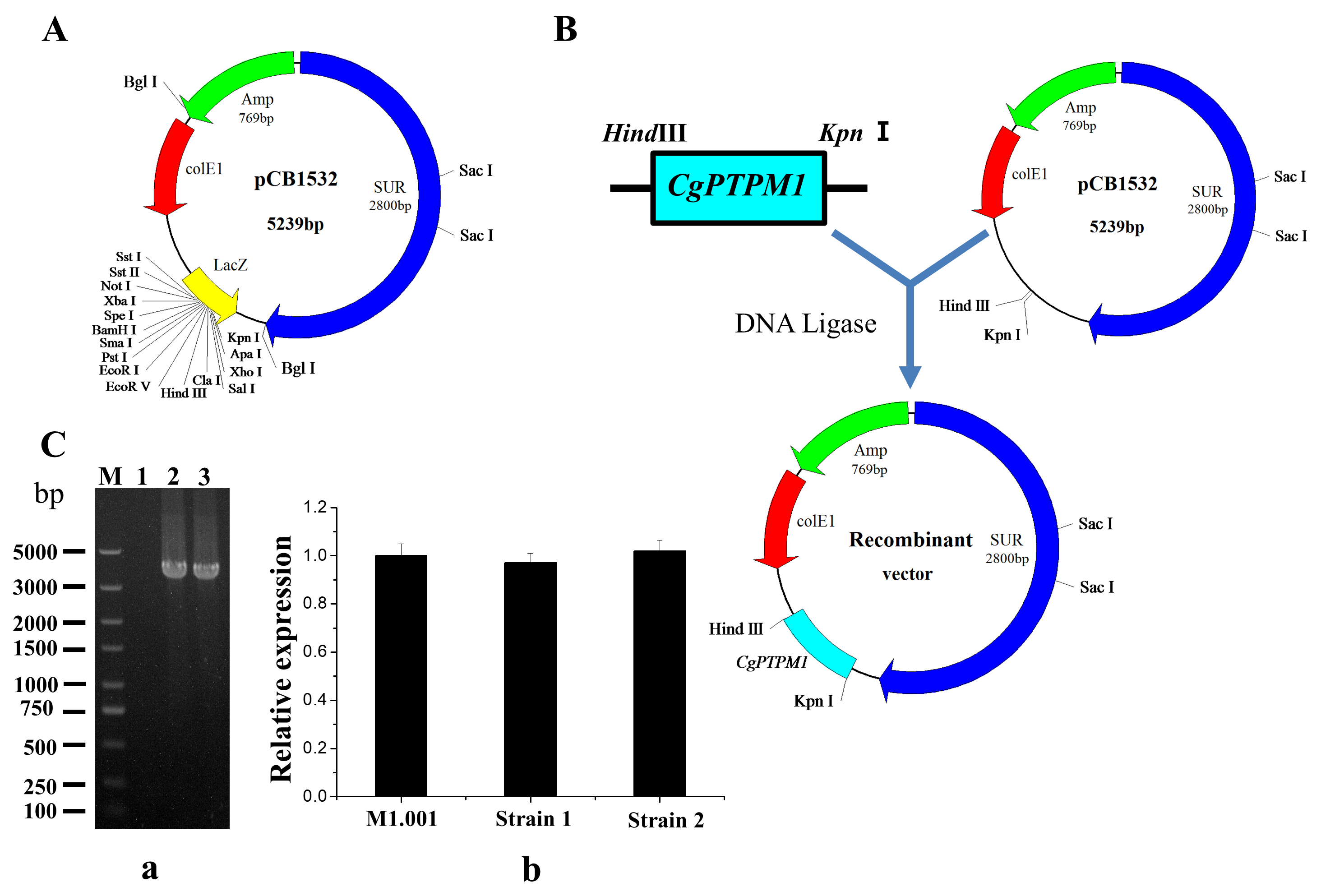

Supplement: Supplementary file 5 [file Image_4.TIF]

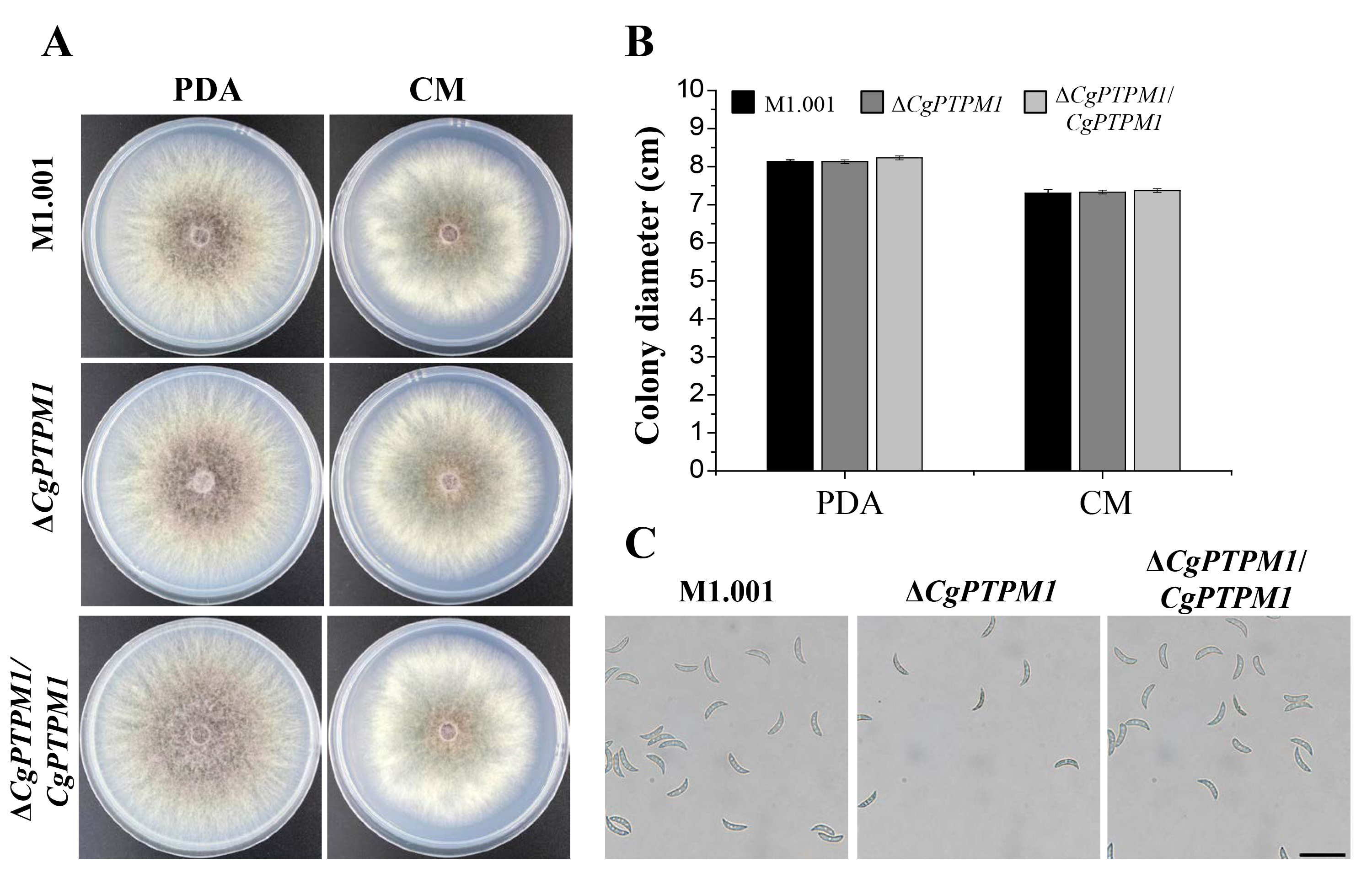

Supplement: Supplementary file 6 [file Image_5.TIF]

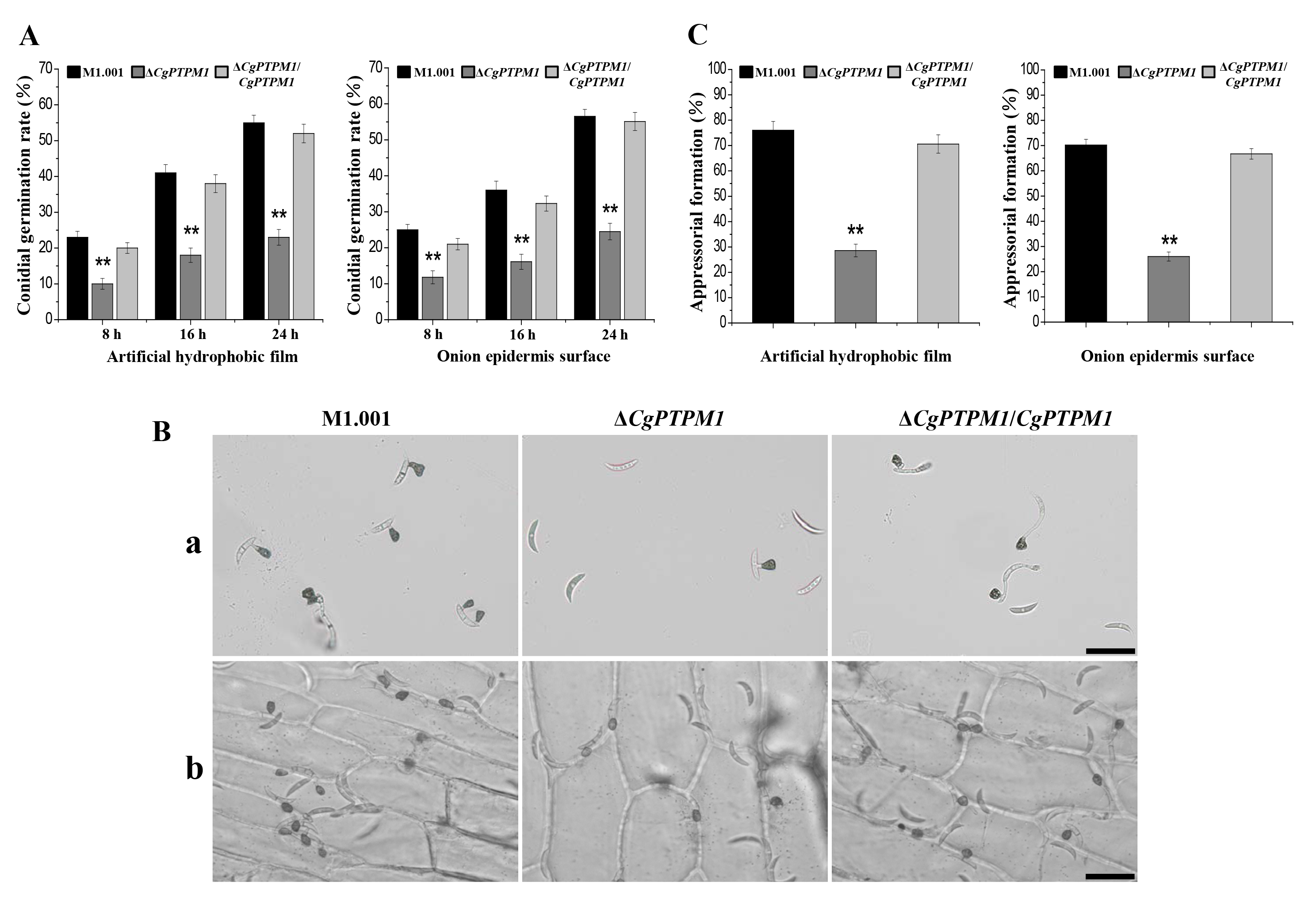

Supplement: Supplementary file 7 [file Image_6.TIF]

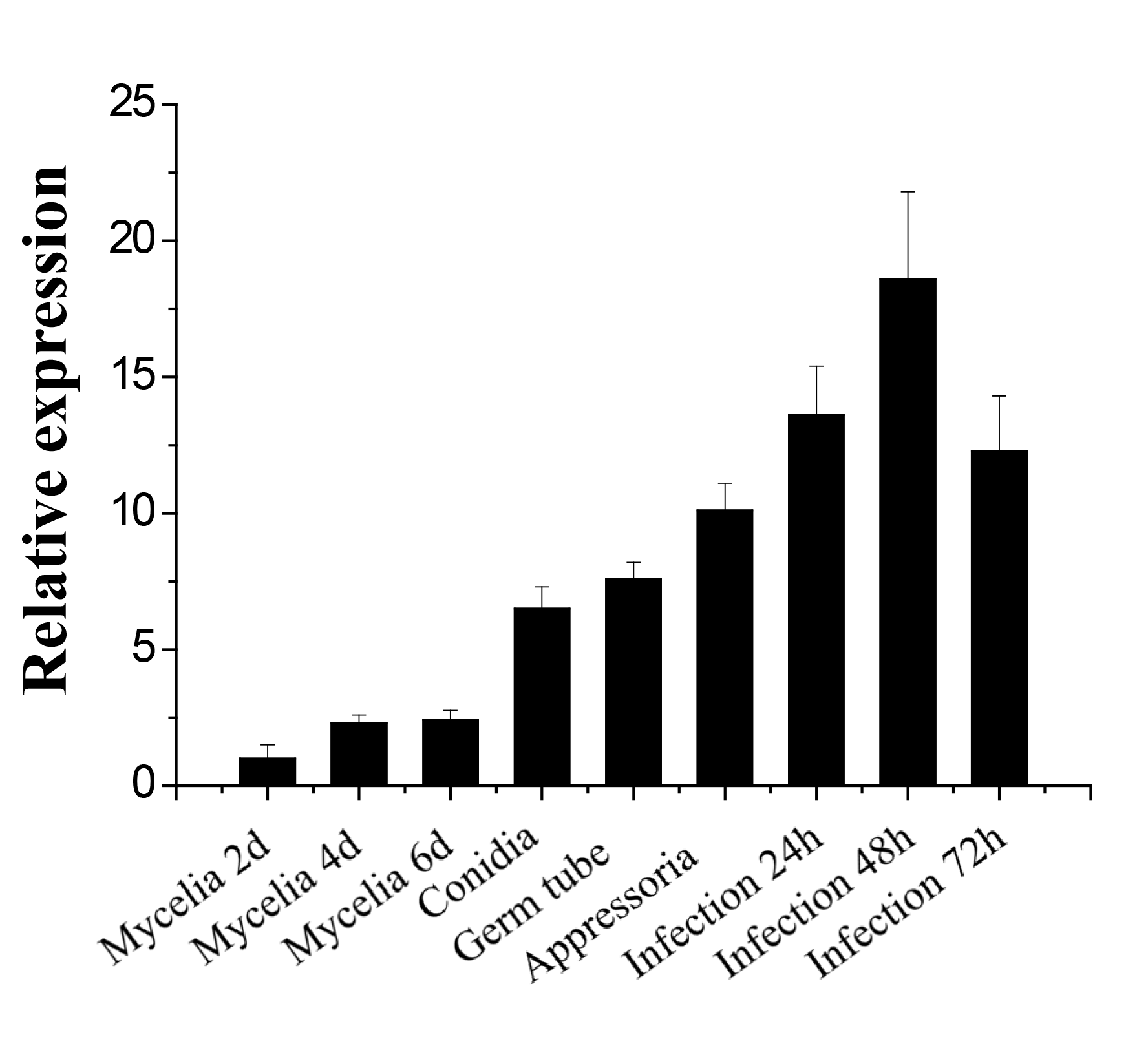

Supplement: Supplementary file 8 [file Image_7.TIF]

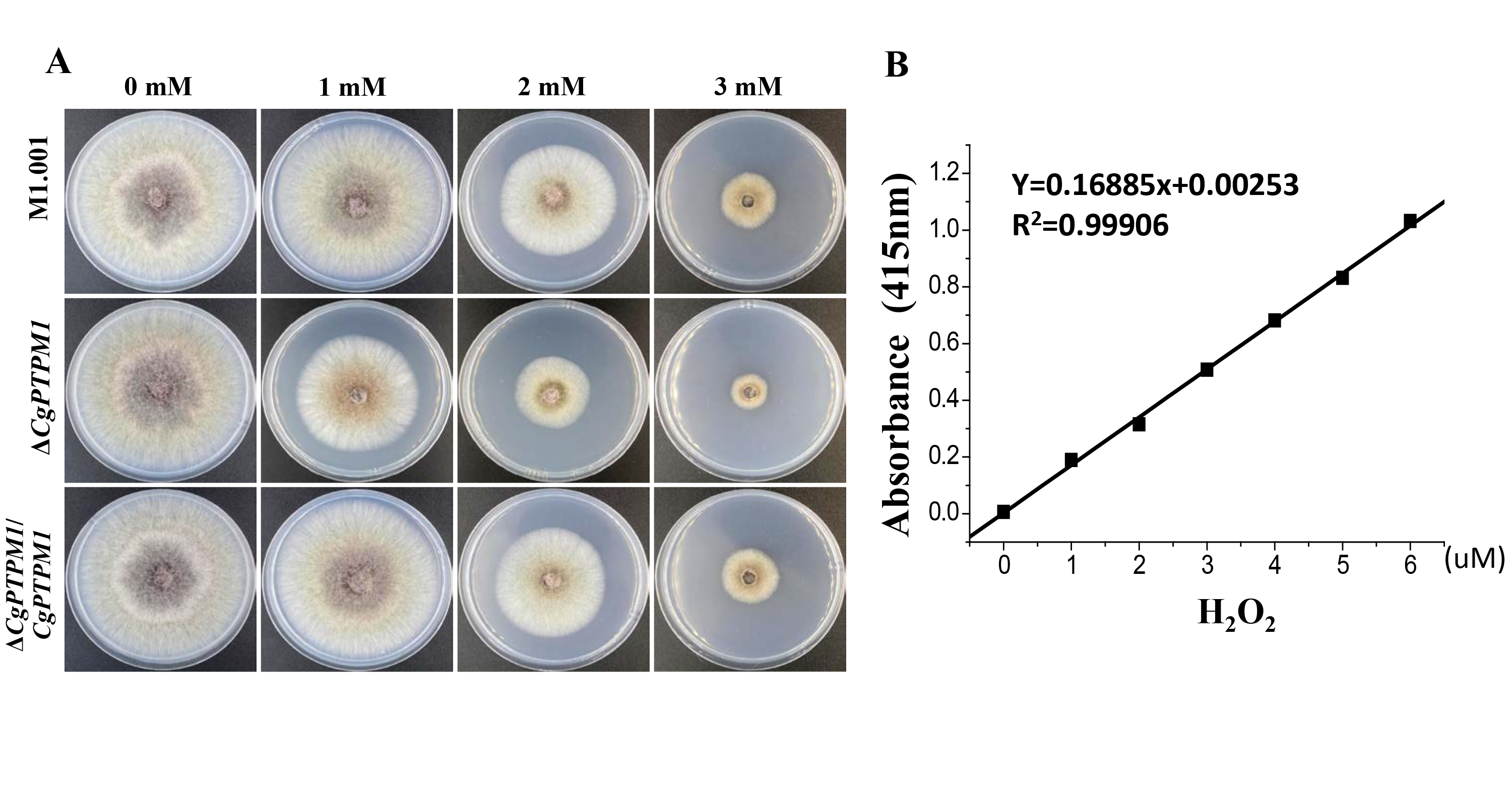

Supplement: Supplementary file 9 [file Image_8.TIF]

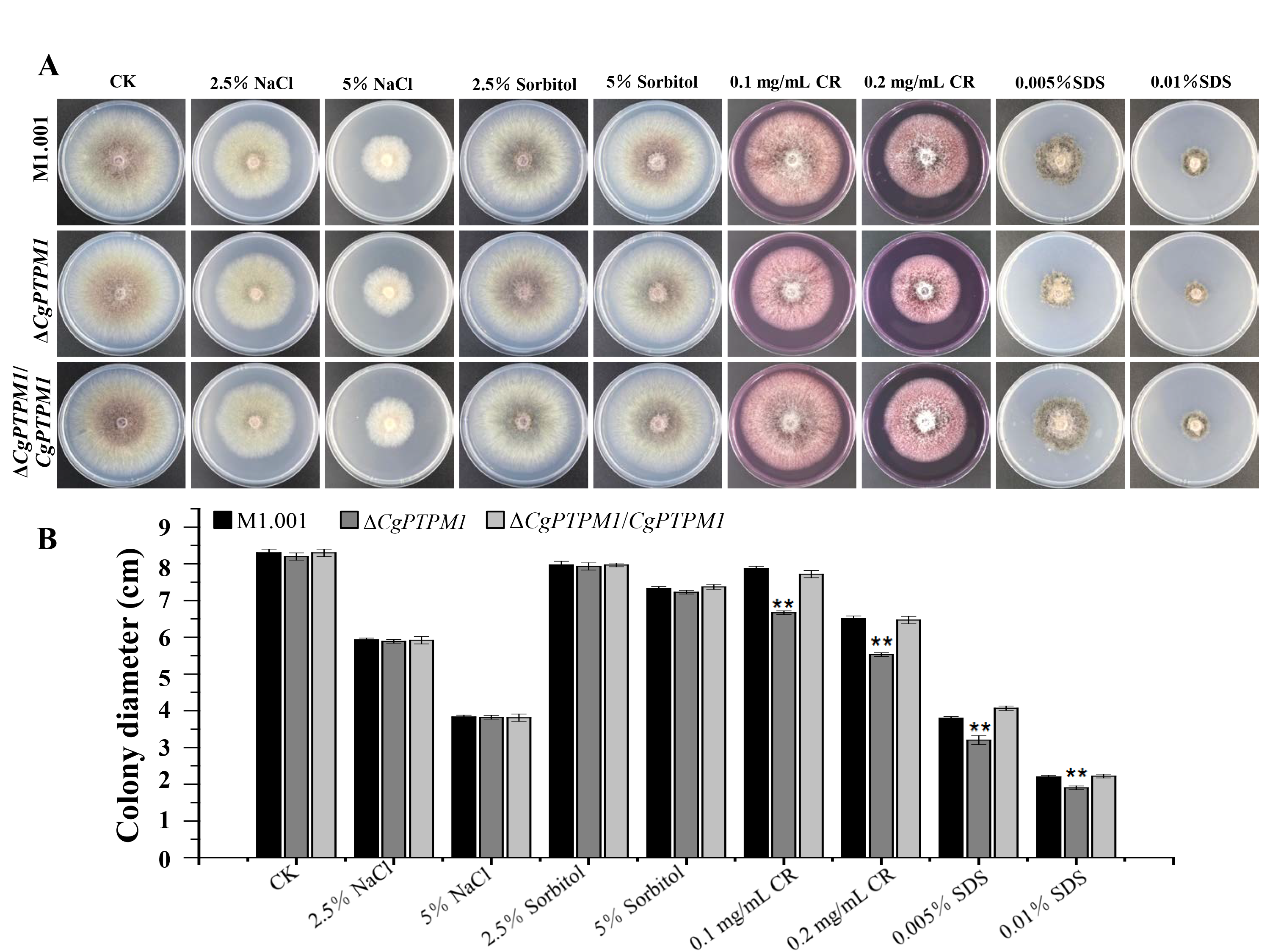

Supplement: Supplementary file 10 [file Image_9.TIF]
